# The Wistar Kyoto Rat: A Model of Depression Traits

**DOI:** 10.2174/1570159X21666221129120902

**Published:** 2023-07-10

**Authors:** Eva E. Redei, Mallory E. Udell, Leah C. Solberg Woods, Hao Chen

**Affiliations:** 1 Department of Psychiatry and Behavioral Sciences, Feinberg School of Medicine, Northwestern University, Chicago, IL, USA;; 2 Department of Pharmacology, Addiction Science, and Toxicology, University of Tennessee Health Science Center, Memphis, TN, USA;; 3 Section on Molecular Medicine, Department of Internal Medicine, Wake Forest School of Medicine, Winston-Salem, NC, USA

**Keywords:** WKY, forced swim test, passive coping, quantitative trait loci, WMI, genetic variants, depression genes

## Abstract

There is an ongoing debate about the value of animal research in psychiatry with valid lines of reasoning stating the limits of individual animal models compared to human psychiatric illnesses. Human depression is not a homogenous disorder; therefore, one cannot expect a single animal model to reflect depression heterogeneity. This limited review presents arguments that the Wistar Kyoto (WKY) rats show intrinsic depression traits. The phenotypes of WKY do not completely mirror those of human depression but clearly indicate characteristics that are common with it. WKYs present despair-like behavior, passive coping with stress, comorbid anxiety, and enhanced drug use compared to other routinely used inbred or outbred strains of rats. The commonly used tests identifying these phenotypes reflect exploratory, escape-oriented, and withdrawal-like behaviors. The WKYs consistently choose withdrawal or avoidance in novel environments and freezing behaviors in response to a challenge in these tests. The physiological response to a stressful environment is exaggerated in WKYs. Selective breeding generated two WKY substrains that are nearly isogenic but show clear behavioral differences, including that of depression-like behavior. WKY and its substrains may share characteristics of subgroups of depressed individuals with social withdrawal, low energy, weight loss, sleep disturbances, and specific cognitive dysfunction. The genomes of the WKY and WKY substrains contain variations that impact the function of many genes identified in recent human genetic studies of depression. Thus, these strains of rats share characteristics of human depression at both phenotypic and genetic levels, making them a model of depression traits.

## 1 INTRODUCTION

In 2016, an editorial in Nature Neuroscience [1] stated, “We will restrict the usage of ‘disease-like’ terms as applied to animal models of psychiatric disease and ask that the particular construct(s) measured be described explicitly.” What followed was a semi-official rejection of preclinical animal work. Defenders of animal models affirmed, “despite the difficulties inherent with modeling brain disorders in animals, when used judiciously—fully cognizant that models of specific behavioral or biological aspects cannot completely recapitulate the human disorder—animal research is crucial for advancing our understanding of neuropsychiatric disease” [2]. Despite this controversy, we purport that animal models of psychiatric diseases do exist and can serve an important purpose in understanding the underlying molecular mechanisms of disease. Specifically, over 600 published articles have used the Wistar-Kyoto (WKY) rat to study depression. Subsequent articles have since identified molecular/genetic markers of disease in this model and demonstrated its translational utility to human depression [3-5].

In this review, we offer the hypothesis that the WKY rat strain is indeed an animal model of depression, presenting the fundamental phenotypes of passive coping and stress hyperreactivity. These phenotypes are coupled with other known dysregulation in major depression, such as circadian rhythms, the hypothalamic-pituitary-adrenal (HPA) and hypothalamic-pituitary-thyroid axes, as well as glucose metabolism. We argue that understanding the pathophysiology underlying the behavioral differences of WKY rats relative to other strains will subsequently shed light on human psychiatric diseases.

### 1.1 History of the WKY Rat

The WKY strain was established at the Kyoto School of Medicine, Japan, from the same parental outbred Wistar stock as the Spontaneously Hypertensive Rat (SHR) strain to serve as its normotensive control [6]. Since the Wistar colony was maintained by outbreeding, and breeding of WKY started later than for SHR, the WKY strain exhibits phenotypic and genetic diversity from the SHR beyond the originally-defined normotensive phenotype. The WKY breeding stock was obtained by NIH from Kyoto (WKY/N) in 1971, and it was subsequently distributed to different vendors prior to being fully inbred [7-9]. For example, Harlan Laboratories (WKY/NHsd) received WKY rats from NIH in 1982, much later than the Charles River Laboratories (WKY/NCrl), which received NIH-WKY in 1971 [7]. Taconic lab received their WKY stock at the 10th generation from the NIH Animal Genetic Resource in 1974 and maintained the colony as outbred. The consequence of the early distribution of WKY breeders to the different vendors is that the WKY substrains differ from each other both phenotypically and genotypically. For example, Pare [9] observed that WKYs from Harlan or Charles River laboratories showed similar stress-responsive and depressive behaviors compared to other strains, whereas the Taconic outbred WKY did not.

## 2 BEHAVIORAL PHENOTYPES OF WKYs

As Sousa and colleagues described in their review [10], behavioral testing seems to be easy, but, in reality, it is a very rigorous and complex process. There are many models used for the study of depressive disorder, including the sucrose preference test (SPT), tail suspension test (TST), and forced swim test (FST). The SPT relies on the long-term preference for sugar solutions and suggests a decreased preference for sucrose is related to anhedonia [11]. Sucrose preference is influenced by both genetic and environmental factors [12-14]. It is often used to measure the hedonic response of animals to a natural reward [15]. The WKY rats exhibit decreased sucrose preference relative to Wistar rats [16]. In contrast to the SPT, the TST and FST are based on the nature of animals to avoid stressful situations. After a certain time, the animal ceases the attempts to escape, and immobility occurs; longer immobility phases are potential signs of depression-like behavior [17]. After the administration of antidepressants, the immobile phase in both the TST and FST is abbreviated, although there are clear strain differences in responsiveness to antidepressants [18]. The TST is mainly used in mice [19], whereas the FST can be used in both rats and mice.

The FST is the most commonly used assay that is thought to measure despair, learned helplessness, or, as commonly implied, depressive behavior. During the FST, an animal is placed in a container filled with water from which it cannot escape. The animal will first try to escape but eventually will stop struggling and exhibit floating/immobility. It is a two-day test where the animal is placed in the water for 15 minutes on day one and 5 minutes on day two. The popularity of this test lies in the effects that antidepressants have on immobility; namely, treatment with antidepressants between days 1 and 2 has been shown to reduce immobility in the FST [19-21].

Despite the frequent use of the FST and its practical value, it is also the most often criticized assay for its “cruelty” as well as whether it measures depressive behavior or other characteristic behaviors [22]. Regarding the claim that the FST is “cruel” to animals, we need to clarify that rats are very good swimmers and live in sewers. Therefore, it is not an unusual activity for them. The fact that they cannot escape from the test is not really different from other behavioral tests, such as learned helplessness or fear conditioning. The claim that immobility is a sign of depressive behavior has been countered by Molejdink and de Kloet [23], who argued that immobility is “a switch from active to passive behavior in the face of an acute stressor, aligned to cognitive functions underlying behavioral adaptation and survival.” Others have also interpreted immobility as passive coping [22, 24]. A very innovative hypothesis suggests that the behavior of animals during the second day of the FST could be the result of two independent factors: the proper consolidation of memory about the prior experience of inescapable stress on day one of the FST, and the individual or strain variation in coping strategy [25].

Pare was the first to demonstrate that WKY exhibited increased immobility in the FST and increased freezing in the learned helplessness (LH) paradigm relative to the Wistar progenitor strain [26]. Since then, multiple laboratories have evaluated the WKYs in the FST; using the key words of WKY and FST, the Google Scholar search resulted in over 1200 publications.

It is argued that two-thirds of individuals with depression also show anxious distress [27], which was added to the diagnosis of depression in the Diagnostic and Statistical Manual of Mental Disorders 5th edition [28]. Thus, increased anxiety is not a necessary symptom of depression but a frequent co-occurring one. Different components of the complex behavioral repertoire of an animal can be measured by behavioral tests that, although thought to measure the same aspect, would differ from each other. Measuring anxiety-like and exploration-based behaviors *via* the open field test (OFT), elevated plus maze (EPM), light-dark test, defensive burying (DB), social interaction, and hyponeophagia tests share some conflict between activity/exploration and avoidance of the novel subject, object, or environment. Anxiety has been evaluated by the time spent in the central part of an OFT or the time spent in the open arms of the EPM, which are often significantly correlated [29]. It is unclear whether the tendency to be active during short exposure (5-10 min) to novel environments is a specific trait of animals distinct from novelty-seeking. The OFT clearly depends on the activity level of the animal, while the exploration of the EPM open arm is often equated with risk-taking behaviors. More anxiety behavior-related tests, such as social interaction, depend on the nature of the conspecifics and their level of threat or attraction; thus, their correlation or lack thereof with other anxiety-related tests is difficult to interpret.

WKY rats show increased anxiety-like behavior in the OFT with decreased time spent in the center zone of the arena but also decreased activity as measured by distance traveled [30, 31]. WKYs cross into the open arm of the EPM but spend less time there compared to Lewis and SD rats [32]. The decreased burying behavior of WKYs in the defensive burying tests is also thought to reflect increased anxiety [33] or passive coping [34] with imminent stress. It also seems to be an avoidant type of behavior, since the animals move to the corner of the cage and remain there without attempting to eliminate the threat as other strains of rats do [35, 36]. Additionally, the NIH-HS heterogeneous stock rats, generated from eight inbred founders, including WKY/N [37], show passive coping responses in the FST and helplessness reactions at the two-way active escape/avoidance task [38]. This further confirms the observations that the predominant behavior of WKYs is to become immobile in the presence of an environmental challenge across different tests. To rephrase the classic “flight or fight” response to stress, the WKY’s response to stress is “freeze or avoid”. Whether this reflects hyperresponsiveness to stress or, rather, a generally passive coping strategy to any change is not clear from these behavioral observations.

Accumulating clinical evidence demonstrates altered sensitivity/tolerance for pain among patients with depression and anxiety [39]. Similarly, WKYs exhibit enhanced nociceptive sensitivity to both acute and persistent stimuli of various modalities. For instance, pain thresholds for cold water stress are reduced in WKY rats compared to SHR [40]. In the hot plate test, WKYs demonstrate significantly shorter pain response latencies compared to the SHR [41] and SD strains [42], indicative of thermal hyperalgesia. WKYs also exhibit exacerbated nociceptive responses to visceral pain [43] and prolonged inflammatory stimuli [42, 44]. Altered pain sensitivity in WKYs compared to other strains is further supported by their hypoanalgesic responsiveness to equivalent circulating plasma levels of morphine [45-47]. These findings are consistent with depression in humans, which is accompanied by pain-related symptoms at an estimated prevalence of 65% [48].

In depressed humans, alcohol use disorder is a common co-occurring condition, likely influenced by the use of drinking as a coping mechanism [49, 50]. The utility of the WKY strain in alcohol research has been overlooked on account of early work showing moderately reduced intake in WKY males compared to other strains [51-53] or no significant strain differences [53]. Based on these male-specific findings, the WKY was rendered a “non-alcohol-preferring” strain [54, 55], thereby discouraging its utility in alcohol research aside from occasional recruitment as a control strain. When used as a control, however, WKY rats often demonstrated increased alcohol consumption compared to other strains, including SD [56], SHR [57], and Wistar [58, 59]. Another study tested the effect of stress on alcohol self-administration in WKY *versus* Wistar rats and revealed exaggerated intake in WKYs both with and without prior stress exposure [60].

Notably, the contradicting reports from early studies were likely influenced by the types of self-administration protocols that were standard during that time, such as limited-access paradigms (*e.g*., daily, 1 h access to alcohol). It is now commonly recognized that these short-access paradigms are not suitable for facilitating the escalation of an intake nor the emergence of group differences. For instance, Soeters *et al.* [57] observed significantly higher alcohol consumption between WKY *versus* SHR males under conditions of continuous (24 h) access to increasing concentrations of alcohol; however, this strain difference was abolished upon subsequently limiting the availability of alcohol to 1 h, daily sessions.

Interestingly, WKYs often demonstrate paradoxical changes in behavior following chronic alcohol exposure compared to other strains. In a study by Pare *et al.* [56], WKY rats that had been previously exposed to alcohol self-administration exhibited significantly less severe stress-induced stomach ulcers compared to those who were alcohol naive. Further, compared to SD rats, WKYs self-administered significantly more alcohol and exhibited anxiolytic responses to chronic alcohol exposure, as measured by altered OFT and EPM behavior [56]. In humans, passive/ avoidant coping is associated with increased alcohol consumption [61] and inflated rates/use of drinking as a coping mechanism for stress and depression management [49, 50]. Thus, increased alcohol consumption in WKY rats under these conditions may be related to the inherent stress reactivity, passive coping tendencies, and/or distinct responses to alcohol exposure of these rats.

Unfortunately, our understanding of alcohol-related sex differences is sorely limited by the paucity of female-inclusive research; however, the increased inclusion of females in recent years has elucidated a trend of exaggerated alcohol consumption in female *versus* male rats and mice [62-64]. Within the WKY strain, few studies to date have probed for differences in alcohol consumption by sex; however, results are consistent in demonstrating significantly enhanced intake among females [65, 66]. These findings are indicative of extensive sexual dimorphism in WKY rats that warrant further investigation. Subsequent work is also needed to re-assess the potential utility of WKYs in addiction research through female-inclusive studies.

## 3 PHYSIOLOGICAL PHENOTYPES

Regulation of peripheral levels of glucocorticoids, thyroid hormones, and glucose is intertwined. Glucocorticoids regulate blood glucose by stimulating hepatic gluconeogenesis, and there is an inter-regulation of glucocorticoids and thyroid hormones at multiple levels of their function. Glucocorticoid and thyroid hormones act *via* their receptors, which are also transcription factors and show synergistic effects in regulating gene expression in different systems [67-69].

### 3.1 The Hypothalamic-Pituitary-Adrenocortical (HPA) Axis

Glucocorticoids are involved in various important biological processes, such as gluconeogenesis, lipid and protein metabolism, anti-inflammatory action, and growth [70, 71], and are the major regulators of the stress response. After exposure to stressful stimuli, cortisol (corticosterone in rodents) is rapidly released from the adrenal glands to provide the energy necessary to respond. Through negative feedback control at the pituitary, hypothalamus, and supra-hypothalamic levels, peripheral glucocorticoid concentrations return to homeostatic basal values after the stressor ceases. In chronic stress, sustained elevations in cortisol have deleterious effects on the organism [72, 73]. Thus, abnormal glucocorticoid levels and dysregulation of the HPA axis are implicated in various pathologies, such as obesity [74], depressive episodes [75, 76], constitutive sensitivity to inflammatory and autoimmune reactions [77], and vulnerability to drug addiction [78].

Several groups have shown that the WKY exhibits altered regulation of the HPA axis relative to other strains [79-83]. Depressed human subjects show significantly higher cortisol awakening responses compared to non-depressed controls [84]. Similarly, WKYs exhibit exaggerated basal levels of plasma ACTH and corticosterone (CORT) at night, which is the active cycle of these nocturnal animals [85, 86]. These elevated basal levels of CORT are in agreement with the enlarged adrenals found in WKY females [86] and WKY males [87] and are consistent with the significantly increased adrenal gland volume in depressed individuals [88]. One can speculate that the elevated basal CORT levels and increased adrenal weight are characteristics of a persistent stress state. Further studies support this hypothesis and suggest a potential causative connection between the “behavioral inhibition state” that the WKYs show and the “persistent stress state” that their HPA function suggests.

If the WKYs are indeed in a persistent stress state, they are expected to show exaggerated HPA responses to acute stress, as described in chronic stress states [89, 90]. In response to acute stress, WKY rats demonstrate greater increases in ACTH relative to other rat strains [32, 79-81] and increased steady-state proopiomelanocortin transcript levels [79, 91, 92]. Plasma CORT responses to acute stress are also exaggerated and/or prolonged in WKYs compared to other strains [79, 91, 93]. Within the WKY strain, stress-induced CORT levels are significantly higher and more variable among WKY females *versus* males [86]. For instance, WKYs respond to acute stress with increased plasma CORT levels, elevated by about two-fold in males [94] and up to five-fold in standardly-housed females [93, 95] relative to baseline. These findings align with evidence from other studies supportive of females having exaggerated HPA axis dysregulation [96]compared to their male counterparts [96].

It is likely that the observed stress hyperactivity of WKYs is due to impaired negative glucocorticoid feedback. In support of this hypothesis, removal of glucocorticoids by adrenalectomy (ADX) has little or no effect on several glucocorticoid-dependent measures in WKY rats, whereas significant changes are seen in the above parameters post-ADX in F344 and Wistar rats [92]. Furthermore, CORT replacement after ADX has no effect on several parameters in WKYs. There is a decreased suppression of CORT after treatment with dexamethasone in WKY rats relative to Sprague-Dawley (SD) rats [79], SHR, and Lewis rats when dexamethasone is given at high doses [97]. This decreased suppression by dexamethasone is similar to the findings in depressed individuals [98-100], further increasing the parallel between HPA abnormalities in depression and that of WKYs. The possible impaired negative feedback in WKY rats does not appear to be due to a downregulation of glucocorticoid receptors (GRs) [81, 82]. In contrast, increased GR staining in the hippocampus of WKY females compared to Wistars, and increased expression of hippocampal *Nr3c1* (GR) in WKYs compared to SD, suggest upregulation of GRs in WKYs [101, 102]. The upregulation of hippocampal GR in the WKYs parallels the antidepressant effects of glucocorticoid synthesis inhibitors and GR antagonists [103-105], while the findings of GR downregulation could be specific to peripheral cells in major depressive disorder (MDD) individuals [99].

The above findings suggest that WKY rats have a decreased sensitivity to endogenous glucocorticoids, which may be causative of the HPA axis hyperactivity to stress in this strain. However, *in vitro* studies show that anterior pituitary corticotropin releasing factor (CRF) binding and CRF receptor 1 mRNA levels are significantly decreased in WKY rats, and the ACTH response to CRF or vasopressin (AVP) is markedly impaired [106]. In contrast, steady-state anterior pituitary POMC mRNA levels are ~12-fold greater in WKY rats compared to Wistar rats, and they further increase in response to CRF stimulation [106]. These findings suggest that, although the WKY corticotroph is under a chronic state of activation or disinhibition, the *in vitro* secretory responses to classic ACTH secretagogues are impaired. Thus, the HPA hyperactivity of the WKY may be due to hyposensitivity to endogenous corticosterone or to decreased inhibition of the HPA function of other origins rather than the result of an increased hypothalamic stimulatory drive.

The HPA response to chronic stress usually depends on whether the chronic stress paradigm employs the same stressor or a variety of stressors [107]. It is thought that the organism can habituate to the same stressor [108]. However, chronic restraint stress still generates elevations in plasma CORT levels that are similar in WKY, Fisher 344 (F344), Brown Norway, and Lewis male rats [94]. The same stress paradigm affects body weight gain less in WKY than in F344 males and generates blood transcriptomic differences between the strains in control and chronic stress conditions [109]. Overlapping differentially expressed genes (DEGs) between control and chronic stress in the blood of F344 and WKY suggest a convergence of stress-related molecular mechanisms independent of stress reactivity. In contrast, DEGs unique to the F344 and the WKY stress responses are divergent in their functionality and networks, beyond that of strain differences in their non-stressed state. Most F344 stress response DEGs overlap with those of WKYs, showing only reduced expressions [109]. In contrast, integrin signaling and the Fc receptor-mediated phagocytosis in macrophages and monocytes are the two canonical pathways characterizing the DEGs unique to WKYs [109]. Both are involved in the removal of pathogens as part of innate and adaptive immunity, and the clearance of apoptotic cells that are generated during development and cell turnover in tissues [110]. Those blood DEGs that overlap with hippocampal or amygdala DEGs obtained in a previous study [94] can mark stress sensitivity and are tested in human blood.

Serum protein levels of human orthologues of these DEGs, in addition to classic stress and general clinical markers, were measured in 33 Battlefield Airmen, during a 52-day long preparatory training course before their course of initial entry [111]. Blood samples and factors of affective state, negative valence “threat”, and positive valence “challenge”, have been obtained across different days of training after either routine physical exercise or prolonged and intense physical and mental training. As expected, serum cortisol levels differed between individuals with the differing success in coping with the strenuous training [111]. Furthermore, serum levels of this stress panel correlated significantly with affective measures after the stressfulness of extended training. This study suggests that a panel of blood markers identified in WKYs can measure stress responsiveness in humans, indicate the affective consequences of stressful events, and has the potential to advance individualized stress-management strategies.

### 3.2 The Hypothalamic-Pituitary-Thyroid (HPT) Axis

The connection between abnormalities of thyroid hormone regulation and mood disorders has been suggested without identifying the molecular cause of this connection [112]. The prevalence of depressive symptoms in hypothyroidism is approximately 60% [113]. Even subclinical hypothyroidism is thought to be a risk factor for depression [114]. Supporting the role of thyroid hormones in depression is the fact that depression and cognitive dysfunction are the most common psychiatric symptoms related to hypothyroidism [115]. Furthermore, the risk factors to develop hypothyroidism include female gender and age, and there are twice as many women with depression as men. However, many studies question the relevance of thyroid function to depression, arguing that the similarities in symptoms do not reflect a cause-and-effect relationship [116, 117]. While a direct relationship between abnormal thyroid function and depression has not been ascertained, the dysregulation of both HPT and HPA axes in individuals with depression suggests that these are either the consequence or a thus far unknown associative cause of depression or, perhaps, more likely, a combination of cause and consequence.

Hypothalamic thyrotropin releasing hormone (TRH) expression is decreased in depressed patients [118]. In agreement with this, a deficient nocturnal surge in serum thyrotropin (TSH) and reduced 24-h TSH secretion were found in depressed individuals compared to healthy controls [119-121]. In addition, the response of TSH to exogenous TRH is blunted in a quarter of depressed patients [122]. These findings suggest reduced hypothalamic drive of the HPT axis, which could be related to enhanced effectiveness of the thyroid hormones-mediated negative feedback. However, while serum thyroxine (T4) and triiodothyronine (T3) levels are in the normal range in depression, the conversion of T4 to the more biologically active T3 is known to be inhibited by higher cortisol [112, 123], and T4 transport across the blood-brain barrier is decreased in depressed patients [124, 125]. Thus, active thyroid hormone concentrations are likely lower in the depressed brain. Based on these findings, both T3 and T4 have been used for the treatment of depression. T3 has been shown to be effective [126, 127] in influencing psychiatric symptoms. The efficacy of T4 in the treatment of bipolar depression has been proven on many occasions [128-132]. This is relevant for the connection between HPT function and depression, as the recent diagnostic manual, the DSM-5, allows for the presence of manic symptoms as part of the depression diagnosis in patients who do not meet the full criteria for a manic episode. Despite the lower nocturnal TSH levels and attenuated TSH responses to TRH stimulation in depressed patients [133], the limited adverse response to supraphysiological doses of T4 in depressed patients suggests a hyposensitivity to thyroid hormones [128, 134, 135].

Investigations of WKY thyroid axis function revealed dysregulations that seem to be different from that of depressed individuals. Abnormally high plasma levels of TSH, despite smaller elevation in serum total T3 levels, compared to Wistars [136], together with elevated TSH levels throughout the 24-hour cycle compared to Wistar and F344 rats [136, 137], could indicate reduced efficacy of thyroid hormones at the level of pituitary. The normal mRNA levels of the preprothyrotropin-releasing hormone, the precursor of TRH, in the WKY hypothalamus in the presence of elevated peripheral T3 also suggest reduced effectiveness of T3 in the negative feedback regulation centrally [138]. Induction of chronic hyper- and hypothyroid states in WKY rats elicits appropriate hormonal responses; however, TSH levels rose higher in response to hypothyroidism in WKY rats than in Wistar controls [136], which is consistent with reduced negative feedback efficacy. Furthermore, behavioral data of WKYs showing resistance to the effects of all but the highest levels of T3 [136] is similar to those in depressed individuals treated with thyroid hormones.

An additional argument for the relevance of abnormal HPT function in depression is the finding that MDD patients exhibit significantly reduced blood levels of transthyretin (TTR), the protein thought to be partially responsible for thyroxine transport across the blood brain barrier [139]. TTR has been recently identified as a novel biomarker not only for MDD diagnosis but also for disease monitoring [139]. TTR is drastically reduced in the hippocampus of selectively-bred Wistar Kyoto More Immobile (WMI) males, who exhibit depression-like behaviors, unlike the control Wistar Kyoto Less Immobile (WLI) rats [94]. Thus, mild HPT abnormalities may be concomitant with increased vulnerability to depression or may be causative of this process. In either case, WKYs and a sub-group of human depressed patients show similarities in their HPT function.

### 3.3 Metabolic Abnormalities

In addition to HPA and HPT abnormalities often found in human MDD, there is also a bidirectional link between MDD and diabetes (both type 1 and type 2), with MDD patients showing higher rates of diabetes relative to the general population [140] and those with diabetes showing increased rates of MDD [141, 142]. The mechanisms underlying this relationship remain unclear. Studies have suggested the role of the immune system [140] and/or the HPA axis [143], but most studies remain associative and lack causal validation. Chronic stress leads to over-activation of the HPA axis, which can precipitate hyperglycemia and increased storage of visceral fat [144]. Chronic stress also activates the autonomic system, which can lead to insulin resistance over time. Both visceral adiposity and insulin resistance are known predisposing factors to diabetes. There is also evidence that both MDD and diabetes are driven by similar underlying genetics [145], which is similar to our studies in the WKY rat [146].

However, non-obese, WKY rats exhibit increased glucose and insulin responses to a glucose challenge relative to several other inbred strains, suggesting they are pre-diabetic [146-148]. This is supported by the fact that they become diabetic when the leptin receptor gene is mutated, causing obesity [149]. Taken together, these results indicate that the WKY rat is a pre-diabetic strain that harbors genetic alleles predisposing to diabetes and further implicates a mechanistic relationship between glucose levels and the HPA axis. These data further indicate that the WKY rat exhibits metabolic abnormalities relative to other rat strains, suggesting a potential link between metabolism and depression in this strain.

## 4 QUANTITATIVE TRAIT LOCI ANALYSES

In an effort to understand the genetic and molecular mechanisms underlying the behavioral and hormonal abnormalities of the WKY rat, we conducted a quantitative trait locus (QTL) analysis using a reciprocal F2 generation of WKY and F344 rats [150-152]. Approximately 500 rats were phenotyped for behaviors in the FST, OFT, and DB, as well as physiological regulation of the HPA and HPT axes and metabolic responses to stress. The phenotypes were chosen first because they appeared to be connected in the literature and second for their significant differences in the parental generation of WKYs and F344s.

At eleven weeks of age, all animals went through the phenotyping protocol. Briefly, animals were weighed and tested in the OFT, followed the next day by the 2-day FST. There was a three-week rest period between the FST and the DB test, during which the animals were left undisturbed. The break was important because the DB test is extremely sensitive to prior stress. A week after the DB test, blood samples were collected *via* the tail cut method for measures of basal and stress CORT, basal TSH, and stress glucose. One week later, animals were fasted overnight to obtain glucose and insulin measures after a glucose challenge. Animals were then weighed and sacrificed by decapitation, and adrenal glands were collected and weighed.

Quantitative genetic analyses were carried out as described [137, 150, 153]. Genome scans were carried out using sex and lineage and a sex-by-lineage interaction as additive covariates. This accounts for average differences among the four groups of rats, defined by the combinations of sex and lineage. Three additional genome scans were run with sex, lineage, and sex-by-lineage, respectively, as interactive covariates. These scans helped us to identify the sex- and lineage-specific effects of QTLs. The logarithm of odds thresholds (established by permutation analysis) is higher for the interactive genome scans because the hypothesis being tested has more degrees of freedom. In each case, we computed the contribution from the interaction term alone and required that this LOD score should exceed a nominal 0.05 threshold in order to declare a significant covariate-by-QTL interaction. These findings were published in several manuscripts, as summarized in Table **1**.

Here we summarize only the significant QTL for all the phenotypes. The QTL analyses of the different traits and specific findings were reported in detail previously [34, 36, 137, 146, 151-153]. What can be seen from the table above is that multiple traits across tests can map to the same locus. For example, both FST climbing and OFT grooming map to rat Chromosome 3, while anxiety behaviors from DB and OFT map to rat Chromosome 6. Although less frequent, there are also examples of behavioral QTL mapping to the same location as physiological QTL. Some of these overlaps are discussed previously [146]. For example, rearing QTL and plasma CORT responses to stress show overlap on Chromosome 2. The connection between HPA regulation and rearing in the OFT has been demonstrated [154, 155], but the overlapping QTL suggests the genetic regulation of these phenotypes. While suggestive QTL are not listed in the table above, the stress glucose loci on Chromosome 5 also overlap a suggestive locus for stress CORT [146, 151]. The cause of these intriguing findings will need to be answered in the future when quantitative trait genes are identified for these phenotypes.

## 5 SELECTIVE BREEDING: WMI AND WLI SUBSTRAINS

During our QTL study, we observed that WKYs show greater variation in the FST than F344 and even some outbred strains such as SD [152, 153]. Based on this observation and that the WKYs may not have been completely inbred at distribution to breeders, we began their selective breeding based on immobility behavior in the FST [156]. The bidirectional selective breeding resulted in the WKY More Immobile (WMI) and the WKY Less Immobile (WLI) strains. WMIs consistently show high levels of immobility [94, 156]. The behaviorally distinct WLI strain was concurrently generated from selecting the mating pairs with the lowest immobility scores. Following selection for generations, the strains were maintained as inbred and, by now, are nearing their 50th generation. Despite their common origin, these strains demonstrate marked behavioral and physiological differences both at baseline and in response to stress.

Consistent with findings in individuals with MDD [157], WMIs display dysfunctions in resting-state hippocampal connectivity [158]. WMIs also show sex differences in the onset of depression and anxiety-like behaviors [159]. Again, depression with comorbid anxiety is more common in women than in men, which is another parallel between this animal model and human depression that could be exploited [160].

Stress during adolescence is thought to be a major contributor to psychopathology in adulthood [161]. In both WMI and WLI rats, a very mild single stress during early adolescence significantly decreases anxiety-like behavior, measured in the OFT, and increases social interaction and recognition in adult males compared to controls [162]. In contrast, no significant effects of early adolescent stress are observed in adult females of either strain in these behaviors. Adult males and females of the genetically less stress-reactive WLI strain show significantly increased immobility in the FST after early adolescent stress, in parallel with the generally accepted consequence of stress during adolescence. In contrast, immobility was not altered in WMI males but significantly attenuated by early adolescent stress in adult WMI females compared to controls; although the cause of this is unknown, one can speculate that the stress-reactive WMI females gained resistance to stress during adolescence. The influence of genetic stress-reactivity differences on the consequences of early life stress does not yet have a parallel in the human pathophysiology literature due to the lack of information on genetic stress reactivity differences in humans.

Post-traumatic Stress Disorder (PTSD) has high comorbidity with major depression (50-84%) [163, 164]. The cause of this high comorbidity might be that the preexistence of depression increases susceptibility to traumatic events [165]. Stress-enhanced fear conditioning is an animal model of PTSD that encompasses both stress-sensitizing effects and conditioned fear memory components of PTSD pathology [166, 167]. If prior stress exaggerates fear memory in one strain of animals but not in another, it may present a model of PTSD that mirrors the variability in susceptibility to PTSD in humans [168]. WMI males show exaggerated fear memory in the contextual fear-conditioning (CFC) paradigm after prior stress, whereas the control WLI males do not. In contrast, female WLIs show this enhanced fear memory, but female WMIs do not [169, 170]. The main findings indicate that the measures of the stress response, percent time spent freezing at the acquisition of the fear conditioning, and the measure of fear memory, which is freezing at recall, differ between these strains in both with and without prior stress conditions. The significant correlations between percent freezing at acquisition and at recall suggest that fear memory differences represent a true phenotype related to the stress-reactivity/passive coping or depressive behavior differences between the strains [169].

Among the modifiable risk factors for cognitive decline with aging is stress, which has also been implicated in the progression of Alzheimer’s Disease (AD) and shown to reduce its age of onset [171]. Stress-related disorders like depression have been linked to a state of accelerated aging, affecting the hippocampus and subsequently promoting pathological cognitive aging [172, 173]. The prevalence of major depression is 32% in mild cognitive impairment and up to 37% in dementia [174]. Even moderate depression increases the risk of progression from healthy to mild cognitive impairment and to dementia [175]. Thus, the findings that middle-aged WMI females show significant impairment in fear memory compared to young WMIs and WLIs and middle-aged WLIs further support that the WMIs are a reliable animal model of stress hyperreactivity and/or depression [170].

This fear memory deficit in middle-aged WMI females is in parallel with decreased expression of hippocampal antioxidant enzymes catalase (*Cat*) and superoxide dismutase 1 (*Sod1*) compared to young animals and same age WLIs [170]. Expressions of *CAT* and *SOD1* are also decreased in postmortem brain regions of individuals with AD and depression compared to those with AD alone, suggesting that the stress-related disorder of depression is indeed a risk factor for AD [176]. Decreased hippocampal expression of the memory enhancer insulin-like growth factor 2 (*Igf2*) and its receptor (*Igf2r*) has also been shown to be reduced in middle-aged WMI females compared to young of both strains [170]. As expected, decreased expressions of *IGF2* and *IGF2R* in the postmortem hippocampus and anterior cingulate cortex are present in individuals with both AD and MDD compared to those with AD [176]. This parallel between aging-induced molecular changes in the brain of the WMI depression model and individuals with both depression and AD further confirms the potential usefulness of this model.

The WMI and WLI strains differ in their brain and blood gene expression profiles. The unique blood and brain genome-wide expression profiles of WMIs compared to WLIs were analyzed in conjunction with another study employing four different strains of rats exposed to chronic restraint stress (CRS) *versus* non-stressed controls [3, 94]. Based on the differentially expressed genes in these two studies, we developed blood transcriptomic markers for MDD, which have shown translational promise [3-5, 177-179]. Blood levels of these transcripts differentiated adolescents with MDD from unaffected controls [3] and distinguished adults with MDD from those with no disorder [4, 5]. Thus, this panel of transcripts may serve as blood markers of MDD in humans and further validate the WMI model.

Within-strain sex differences are also present in baseline and stress-induced levels of plasma CORT. Resting levels of plasma CORT are significantly higher in WMI females compared to WLI females, whereas this strain difference is either absent or shows the opposite pattern in males [180, 181]. The inverse of this latter pattern is also present in steady-state hippocampal glucocorticoid receptor (*Nr3c1*) expression [181]. Interestingly, female WMIs and male WLIs do not show a significant increase in plasma CORT in response to restraint stress, as seen in female WLIs and male WMIs. Hippocampal *Nr3c1* expression changes in response to stress are also the opposite in WMI males and females [181].

Another characteristic of human depression is altered tolerance or sensitivity to pain [39]. Compared to SD controls, both WMI and WLI rats exhibit significantly lower thermal pain thresholds when tested in the tail immersion assay [182]. These findings are consistent with human MDD patients, who demonstrate reduced thresholds for pain relative to non-depressed controls [183]. The study by Udell *et al.* [182] further elucidated significant differences in pain sensitivity within the WKY substrains, such that latencies to elicit a pain response were significantly shorter among WMIs compared to their depression-resistant WLI counterparts. The increased pain sensitivity observed in WLI *versus* SD rats is likely related to their anxious phenotype, as the severity of anxiety symptoms predicts self-reported pain perception ratings in humans [184]. Together, these findings support the utility of the WKY and WMI/WLI substrains for studying the relationship between depression and sensitivity to nociceptive stimuli.

Both affective disorders and chronic pain are known to modulate vulnerability to opioid addiction [185]. In a recent study [186], oral self-administration of oxycodone was significantly enhanced in WMI females compared to WLI and Lewis rats, which readily self-administer various drugs, including opioids [187]. Within the WMI strain, females exhibit significantly higher oxycodone intake relative to males; this sex difference was also observed in Lewis rats, although the effect did not reach significance in WLIs [186]. Similarly, WMIs self-administered greater amounts of alcohol than WLI rats after ten consecutive 1 h sessions [181]. Notably, limited-access conditions are often insufficient for detecting strain differences or observing escalation of intake. Nonetheless, WMI rats significantly escalated their intake across sessions, unlike their WLI counterparts [181]. Additional work is needed to evaluate the potential utility of WMIs as models of comorbid alcohol use disorder and depression. This high phenotypic variability between and within (sex differences) the isogenic, WMI/WLI substrains [188] underscores their broad utility within and beyond the fields of nociception and addiction.

Decades of research have established significant variability in the above phenotypes by sex as well as various strain-by-sex interactions. Thus, the substantial phenotypic variability yet highly conserved genetics between WMI and WLIs underscores their value for identifying specific molecular determinants of phenotypic variation, such as in conferring stress vulnerability *versus* resilience, propensity for developing depression, substance abuse potential, and so on. The sex differences within and across the strains emphasize the genetic epigenetic interactions, where the latter is generated by sex hormones. Since many of the human traits that this animal model mirror are also sexually dimorphic, they could also be highly useful in exploring these interactions and learning of their significance.

## 6 GENETIC VARIANTS IN WKY AND RELATED STRAINS ARE IMPLICATED IN HUMAN MDD

Unique genetic variants are the foundation of the various phenotypes of the WKY rats discussed above. Whole genome sequencing data for WKY and related strains have been generated by several studies. Both Ramdas *et al.* [189] and Baud *et al.* [190] sequenced the eight founder strains of the heterogeneous stock rats, including WKY/N. Atanur *et al.* [191] sequenced the genome of 25 inbred strains of rats. These included three WKY substrains: WKY/NHsd, WKY/ NCrl, and WKY/Gla (from Glasco). In addition, de Jong *et al.* [188] sequenced the WLI and WMI strains. While the results described in the original publications were analyzed using an older version of the rat reference genome, we obtained the original sequencing data and reanalyzed them against the latest mRatBN7.2 [192] reference. We did not include the data by Baud *et al.* [190] in this reanalysis because it was obtained using SOLiD, a sequencing technology not comparable with current analysis tools. We used deepvariant and GLNexsus [193] to call the variants, and also used the SNPrelate [194] package to generate a phylogenetic tree. As shown in Fig. (**1**), the WLI/WMI rats are more closely related to WKY/N, reflecting the history of their origin and selective breeding. Between the commercially available strains, WKY/NHsd is more closely related to WKY/N than WKY/NCrl. Unfortunately, the WKY/NHsd was sequenced at only about 11X coverage, which makes this result tentative. On the other hand, the sequencing data from WKY/N had about 30X coverage of the whole genome. We therefore annotated the functional consequence of single nucleotide polymorphisms (SNPs) in WKY/N. We identified 1053 variants predicted to have a high disruptive impact on 848 proteins, such as by causing protein truncation, loss of function, *etc*. These variants are provided in Supplementary Table **S1**. We subsequently searched the human genome-wide association study (GWAS) catalog [195], downloaded on 07/09/2022, and found that 18 of these disrupted genes have already been associated with depression with genome-wide significance (Table **2**). Using the *GeneCup* search engine [196], we found that eight of these genes (*ASIC2, AIF1, CACNA1C, COP1, MICB, MSRA, SHANK2,* and *WNT3*) have well-defined biological functions relevant to depression. For example, the *CACNA1C* gene encodes the L-type Ca^2+^ channel Cav1.2 subunit. The WKY/N genome contains a variant that affects the splicing of the *Cacna1c* gene. The heterozygous deletion of *Cacna1c* results in a mitigated depression-like phenotype compared to the wildtype mice [197], which was further confirmed by viral vector-mediated deletion of *Cacna1c* in the prefrontal cortex [198]. *Shank2* (splice donor variant in the WKY/N genome) is an abundant postsynaptic scaffolding protein that is involved in synaptic plasticity [199] and has been associated with several mental disorders, such as autism, schizophrenia, and neurodevelopmental disorders [200, 201]. Interestingly, several of the disrupted genes, including *Cop1* (frameshift variant), *Msra* (splice donor variant), and *Micb* (stop lost variant), share common involvement in the oxidative stress pathway [202-204], including in the hippocampus [205]. This is in line with emerging evidence from human studies suggesting the role of oxidative stress in disrupting synaptic function [206] and the etiology of depression [207-209]. These data provide strong evidence that the WKY/N, the parental strain of the commercial WKY rat, contains mutations in key proteins associated with major depressive disorder in humans. These compelling genetic data further support the validity of the WKY as a genetic rat model of depression.

In work by de Jong *et al.* [188], the WLI and WMI genomes were sequenced using three different technologies, including Ion Torrent, Illumina, and 10X genomics linked read, to a total of about 100X coverage. Approximately 4, 000 high-quality, homozygous SNPs and indels segregated between these two strains, thereby providing a limited space for identifying genetic variants driving the differences in their phenotype. Some candidate genes were suggested, such as *Gnat2*, *Prlr*, *Nlrp1a*, *etc*. [188]. The limited difference between these two WKY substrains provides an excellent opportunity to use the reduced complexity cross strategy [210] for genetic mappings on phenotypes different between them, including behaviors related to not only depression but also stress reactivity, aging, pain, or substance abuse.

## CONCLUSION

**Fig. (2):**
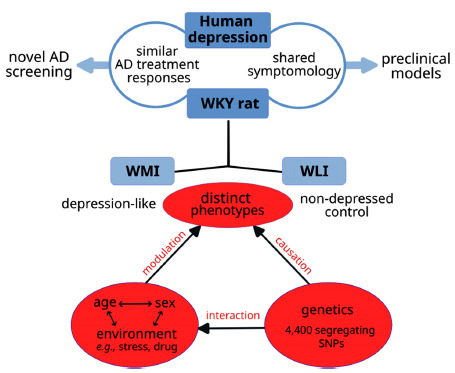
Translational utility of the WKY rat and selectively-bred WMI/WLI substrains. The WKY captures an array of behavioral and physiological traits that are characteristic of human depression. Importantly, the WKY expresses an endogenous depression-like phenotype, unlike environmental models that require the induction of these behaviors by stress. WKY exhibits reductions in depression-like behaviors following treatment with various classes of antidepressants (ADs), such as SNRIs, tricyclics, MAOIs, and ketamine. However, WKY rats lack responsiveness to SSRIs and can be used to identify novel therapies for treatment-resistant depression, which affects a considerable number of depressed individuals. The selectively-bred WMI and WLI rat substrains exhibit extensive phenotypic differences yet highly similar genetics (only differ by ~4, 400 polymorphisms). This underscores their utility for studying the genetic/molecular determinants driving any of their many intrinsic phenotypic differences, such as stress reactivity, depression propensity, substance abuse potential, pain sensitivity, and so on. The WMI/WLI strains offer additional value for studying various gene by environment interactions (*e.g*., exposure to different types of stressors, drugs, novelty, social environments, *etc*.) as well as interactions between the environment and biological factors, such as sex and aging.

This review demonstrates and confirms the uniqueness of the WKY rat strain as a genetic model of passive coping/depression that shows symptoms in parallel with depression in humans. Together with the two selectively bred substrains, these rats offer great translational value for studying human depression and testing novel therapeutics (Fig. **2**).

Table **3** [228-240] summarizes the current diagnostic criteria for MDD and compares that to WKY phenotypes. Specifically, according to DSM-5, either depressed mood or anhedonia plus five of the other symptoms have to be present for over two weeks for the diagnosis of depression. Although it is not possible to ask if the rat is feeling depressed, all other measurable categories of variables of human depression are present in the WKYs. In addition to these behaviors, the WKY rat has several physiological features (*e.g*., altered HPA/HPT and metabolism) that are also seen in human depression. Finally, the fact that the WKY rat harbors genetic variants within genes that are associated with depression in humans further argues the validity of this animal model.

Given the evidence of multiple behavioral and physiological measurements coupled with genetic variants, it is hard to argue that the WKY rat is NOT a model of depression. In contrast, others argue that the FST is not a measure of depression-like behavior but, rather, of stress coping strategy [22, 211, 212] and that the learned helplessness test measures the same [213]. Thus, WKY is a strain showing either depressive or passive coping behavior. This conundrum seems to be an artificial one and perhaps based on the argument that animals cannot be depressed but can show passive coping. However, the fact that animals can show behaviors that resemble depression, grief, and other emotions has been long discussed and beautifully illustrated in The Expression of the *Emotions in Man and Animals* by Darwin [214], in which he argued that all humans, and even other animals, show emotion through remarkably similar behaviors. In an elegant study, Dolensek *et al.* [215] identified facial expressions as “innate and sensitive reflections of the internal emotion state in mice”. Then, they identified that the activity of single neurons in the insular cortex, a brain region relevant to human affective states, correlates with facial expression in the mouse. As time goes on, it is clear that emotion and affective states are not unique to humans but can be ascertained and studied in animals.

There are many animal models that show behavioral characteristics similar to human depression. Aside from transgenic models, where a single gene is modified, stress-induced depression models are very common [216]. Both early-life and adulthood exposure to stressors, such as chronic unpredictable mild stress [217] or social defeat stress [218], generate vulnerability in animals that can parallel those of human depression when it is induced by stressful life events [219]. However, it is not clear how long the depression traits last after the cessation of these stress paradigms; thereby, the major value of these models is that they are easily accessible. Other multigenic models, including those that are either generated by nature, such as the WKY, or by selective breeding, *e.g*., the WLI and WMI [156], the Flinders-sensitive line (FSL) rats [220], the selectively-bred high and low responders (bHR and bLR) [221], or the Roman high- and low-avoidance (RHA/Verh and RLA/Verh) [222], show some similarities and differences in their depression-like traits [223, 224]. Notably, the avoidant or socially inhibited type of depressive-like behavior is seen most clearly in the WKY strain compared to FSL rats, both in adulthood and in prepubertal adolescence [224, 225]. The bLR animals show attenuated extinction in fear conditioning, suggesting that they are more likely a model of post-traumatic stress disorder [226]. The RHA/Verh rats seem to model behavioral disinhibition, including novelty seeking and vulnerability to drug addiction [227]. As described above, WKYs (and the WMI) also show vulnerability to drug addiction, but the behavioral pattern shown throughout development is best described as the manifestations of depression traits.

Additional arguments for the WKY showing trait characteristics and treatment responses similar to depression are plentiful. The WKYs seem to be resistant to the antidepressant effects of selective serotonin reuptake inhibitors (SSRIs), and many depressed individuals are also non-responsive to SSRI treatment [241-243]. Thus, SSRI resistance cannot eliminate the WKY’s status as an animal model of depression traits. Further, a recent systematic review argues that there is no consistent evidence for an association between serotonin and depression and no support for the hypothesis that depression is caused by lowered serotonin activity or concentrations [244]. Therefore, SSRI resistance, whether in MDD or its proposed animal models, does not preclude depression traits or states. Importantly, WKYs do show reduced despair-like behaviors in response to treatment with some classical or novel antidepressants (for review, see [18]). In addition, electroconvulsive stimulation, the equivalent of electroconvulsive treatment in humans, does reduce WKY immobility in the FST without increasing general activity measures [245, 246]. Other therapies that reduce anhedonia in stressed WKYs include deep brain stimulation [234, 247, 248] and low doses of ketamine [234, 249-251], which have shown similar clinical efficacy in treating pharmacoresistant MDD patients [252, 253]. We do not know if these treatments in humans generate active coping or reduce passive coping with stress, but we know that they are effective in reducing depressive symptoms. Therefore, calling the WKY strain an endogenous model of depression, and apparently, one that is resistant to SSRIs [18, 254], is practical, particularly in search of novel treatments.

Additionally, gene expression differences between the two substrains, the WLI and WMI, have contributed to identifying blood-based transcriptomic markers that could distinguish individuals with MDD from those without [3, 5]. These studies were carried out blindly and showed a high level of specificity [179]. Another study in pregnant women with prior and current depression indicates that some of the identified transcriptomic markers can also serve as measures of depression severity [4]. Thus, regardless of whether these selectively bred strains differ in their despair-like behavior in the FST or by their coping strategy in this test, they clearly mirror some components of human depression.

These studies have also posed other questions: Is stress reactivity a general vulnerability factor for behavioral changes associated with depression as well as for drug use? Can stress reactivity be altered by resilience training? Can QTL between WLI and WMI identify some relevant genes for stress reactivity?

An accepted theory for the etiology of depression is the diathesis-stress model, which states that stress may activate an existing vulnerability, transforming the potential of predisposition into the actuality of psychopathology. Although this theory does not take into consideration whether existing vulnerability to stress can generate a synergistic interaction with vulnerability to depression, some studies suggest that is the case [255]. Since stress and depression are known to play a role in drug use and abuse [256-259], the same synergistic interaction between existing vulnerability to drug use, stress, and depression may also be present. This would suggest that resilience training to stress could reduce both depression and drug use [260, 261]. These possibilities could eventually be explored using an animal model that shows all three traits: genetic vulnerability to stress, depression and addiction. In an animal model, causative genes and pathways could also be identified. Once identified, the expression of causative genes can be altered in a brain-specific manner during development or adulthood, followed by in-depth behavioral and physiological studies that would not be possible in humans. We, therefore, propose that the WKY and its substrains, the WLI and WMI, are animal models invaluable to the identification of the intertwined neurobiological and neurogenetic mechanisms underlying stress reactivity, depression, and more.

## Figures and Tables

**Fig. (1):**
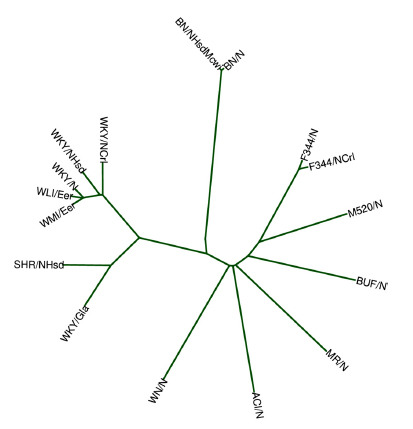
Phylogenetic tree of several inbred strains and substrains of rats. The tree was generated based on the dissimilarity in the genetic variants between the strains. The lengths of the linear path between strains are indicative of their genetic differences. Detailed methods are described in the main text.

**Table 1:** Significant QTLs for All phenotypes.

Phenotype (Test)*	**Markers (cM)**	**Location (mRatBN7.2)**	**LOD**	**%var**	**References**	**Names**
Approach (DB) e	D1Rat145(35cM)	Chr1: 269901560 - 269901692 (Rnor_5)	6.13	6.2	Ahmadiyeh *et al.*, 2005	Stresp9
Glucose (GTT) c			4.91	10	Solberg Woods *et al.*, 2009	Niddm1
Glucose, postrestraint a			4.27	-		Gluco45
Insulin (GTT) a			4.47	-		Niddm35
Body weight a			4.68	-		Bw85
Climbing (FST) b	D1Rat147(100cM)	Chr1: 190409744 - 190409955	-	2.7	Solberg *et al.*, 2004	Despr9
Latency (DB) d	D2Rat188(1cM)	Chr2:144914566 - 144914751	4.56	4.9	Ahmadiyeh *et al.*, 2005	Stresp13
Climbing (FST) b	D2Rat220(60cM)	Chr2: 137154180 - 137154326	-	7.8	Solberg *et al.*, 2004	Despr10
Adrenal weight b			10.2	7.2	Solberg *et al.*	Sradr2
Rearing (DB) e	D2Rat236 D2Rat139 (71-95.4cM)	Chr2:196204417 - 196204629 Chr2: 223490383 - 223490532	6.4	11.75	Baum *et al.*, 2006	Anxrr18
CORT10 b			3.7	1.1	Solberg *et al.*, 2006	Srcrt1
Climbing (FST) d	D3Rat181 D3Rat71 (40-55cM)	Chr3:50968507 - 50968636 Chr3:119584396 - 119584532	4.34	4.8	Solberg *et al.*, 2004	Despr11
Grooming(OFT) a			5	2.9	Baum *et al.*, 2006	Despr2
Body weight a	D4Rat115 (28cM)	Chr4:37177950 - 37178196	4.78	-	Solberg-Woods *et al.*, 2009	Bw86
Adrenal weight b	D4Rat128(38cM)	Chr4: 79986873 - 79987019	6.2	3.2	Solberg *et al.* 2006	Sradr3
Glucose, post-restraint	D5Rat131 (23cM)	Chr5: 39377361 - 39377516 (Rnor5)	3.86	-	Solberg *et al.*, 2009	Gluco47
Approach (DB) e	D6Rat46(1-29cM)	Chr6: 5074497 - 5074641	-	6.8	Ahmadiyeh *et al.*, 2005	Stresp10
Latency (DB) b			3.55	3.5	Ahmadiyeh *et al.*, 2005	Stresp10
Rearing (OFT) a			11.1	8.9	Baum *et al.*, 2006	not registered
TSH a	D6Rat103(55cM)	Chr6: 100364669 - 100364901		8.4	Baum *et al.*, 2005b	Tshhl1
CORT10 b	D6Rat111(74cM)	Chr6: 121497441 - 121497700	6.4	3.9	Solberg *et al.* 2006	Srcrt4
Adrenal weight e	D7Rat24(56cM)	Chr7:66281598 - 66281749 (Celera)	5.6	3.1	Solberg *et al.* 2006	Sradr5
Shocks (DB) d	D7Rat68(80cM)	Chr7: 112982090 - 112982302	4.37	3.8	Ahmadiyeh *et al.*, 2005	Stresp8
Approach (DB) e	D8Rat66(60cM)	Chr8: 95972995 - 95973095	6.83	6.9	Ahmadiyeh *et al.*, 2005	Stresp11
Fasting glucose	D8Rat43(40cM)	Chr8:48630131 - 48630243	3.95	-	Solberg Woods *et al.*, 2009	Gluco43
Rearing (OFT) b	D10Rat153(51cM)	Chr10: 73103259 - 73103421	5.07	3.2	Baum *et al.*, 2006	Anxrr19
Insulin (GTT) c	D12Rat69 (18cM)	Chr12:19950432 - 19950632	4.98	-	Solberg Woods *et al.*, 2009	Insul14
Body weight	D13Rat26 (22cM)	Chr13: 22692481 - 22692658	6.04	-	Solberg Woods *et al.*, 2009	Bw89
Approach (DB) b	D13Rat77(30cM)	Chr13: 60933298 - 60933518	3.35	3.4	Ahmadiyeh *et al.*, 2005	Stresp12
CORT10 b	D15Rat50(64cM)	Chr15: 98596545 - 98596747	3	2.8	Solberg *et al.* 2006	Strcrt5
Climbing (FST) e	D16Rat75(20cM)	Chr16: 22477482 - 22477621	7.49	5.1	Solberg *et al.*, 2004	Despr7
Adrenal weight	D18Rat96(16cM)	Chr18: 39621339 - 39621586	5.8	1.4	Solberg *et al.*, 2006	Sradr6
Body weight	D18Rat121(44cM)	Chr18:71019930 - 71020158	3.54	-	Solberg Woods *et al.*, 2009	Bw92
Latency (DB) a	DXRat82-DXRat67 (5-15cM)	ChrX:19620452 - 19620656(Celera) - ChrX:41304447 - 41304683	4.96	6.7	Ahmadiyeh *et al.*, 2003	Stresp1
Duration (DB) a			4.22	6.2	Ahmadiyeh *et al.*, 2003	Stresp1
Insulin (GTT) c			4.2	16	Solberg Woods *et al.*, 2009	Niddm16 o
Approach (DB) b	DXRat127(25cM)	ChrX: 100567703 - 100567836	3.4	4.9	Ahmadiyeh *et al.*, 2003	Stresp2
Approach (DB) d	DXRat104(45cM)	ChrX: 136437741 - 136437891	4.61	7.7	Ahmadiyeh *et al.*, 2003	Stresp3

**Note:** LOD scores are not comparable across different models due to differing degrees of freedom. *Best covariate mainscan model: a, unadjusted; b, additive; c, sex interactive-lineage additive; d, sex additive-lineage interactive; e, sex and lineage interactive. The genome-wide significance threshold for main effects (including additive and dominant components, 2df) is 3.2-3.6; for QTL interacting with a covariate, 4-4.2 (4df LOD) or 6-6.2 (8df LOD). Names from Rat Genome Database (RGD) unless otherwise specified.

**Table 2:** Genes with high impact variants in the WKY/N genome that have been associated with depression in humans.

**Gene**	**Trait**	**SNP**	***P*-value**	**PMID**
AIF1, BAG6, LY6G6E, MICB	Asthma and major depressive disorder	rs2855812	2.00E-16	31619474
ASIC2	Major depressive disorder	rs387627	2.00E-09	34045744
BTN3A2	Depression	rs13218591	2.00E-08	29942085
CACNA1C	Bipolar disorder or major depressive disorder	rs1006737	3.00E-08	20351715
CACNA1C	Major depressive disorder and other psychiatric disorders (combined)	rs1006737	5.00E-09	23453885
CACNA1C	Broad depression or schizophrenia	rs10774037	8.00E-12	30626913
CACNA1C	Broad depression or bipolar disorder	rs4765914	5.00E-09	30626913
CACNA1C	Major depressive disorder and other psychiatric disorders	rs4298967	8.00E-20	31835028
CACNA1C	Major depressive disorder and other psychiatric disorders	rs10491964	2.00E-08	31835028
CACNA1C	Bipolar disorder or major depressive disorder	rs769087	3.00E-08	31926635
CCSER1	Major depressive disorder	rs10026036	4.00E-09	34045744
CDH9	Depression	rs1946473	3.00E-08	29942085
CDH9	Major depressive disorder	rs10805794	2.00E-08	34045744
COP1	Depression	rs10913112	2.00E-20	30718901
COP1	Bipolar disorder or major depressive disorder	rs10913112	1.00E-10	31926635
COP1	Endometriosis or depression (pleiotropy)	rs6680839	9.00E-10	32959083
COP1	Endometriosis or depression (pleiotropy)	rs2175177	1.00E-08	32959083
LACC1	Depression	rs4143229	3.00E-08	29700475
LETM2	Major depressive disorder and other psychiatric disorders	rs4647903	2.00E-09	31835028
MSRA	Depression	rs17708090	1.00E-13	33859377
MSRA	Depression	rs34328494	1.00E-09	33859377
MSRA	Major depressive disorder	rs67455183	2.00E-10	34045744
PIPOX	Depression	rs75581564	3.00E-13	30718901
PIPOX	Bipolar disorder or major depressive disorder	rs75581564	2.00E-10	31926635
PIPOX	Major depressive disorder	rs75581564	7.00E-10	34045744
SHANK2	Depression	rs7117514	4.00E-14	30718901
SZT2	Major depressive disorder and other psychiatric disorders	rs2842198	1.00E-08	31835028
UBA7	Depressed affect	rs3819325	3.00E-08	29942085
WNT3	Depressed affect	rs199505	1.00E-18	29942085

**Table 3:** Comparison of depression diagnostic criteria and WKY behavioral patterns.

**DSM-5 Criteria**	**Behavioral Parallel**	**WKY**
Depressed mood	NONE	-
Anhedonia	Sucrose*, saccharin consumption/preference, Hyponeophagia* Decreased mating success, Social avoidance	De La Garza *et al.*, 2005 [228] Wrights *et al.*, 2020 [229] Pardon *et al.*, 2002 [32] Burke *et al.*, 2016 [230] Pare, 1994 [231] Ferguson & Cada, 2004 [232] Pare, 2000 [233]
Weight loss/gain	Weight difference	Burke *et al.*, 2016 [230] Willner, 2019 [234]
Sleep disturbance	Sleep disturbance	Dugovic *et al.*, 2000; [235] DaSilva *et al.*, 2011 [236] Ivarson *et al.*, 2005 [237]
Psychomotor retardation or agitation	Freezing/immobility in OFT, FST, DB, LH	Pare, 1993; 1994 [35, 80] Baum *et al.*, 2006 [36] Solberg *et al.*, 2003 [86] Ahmadiyeh *et al.*, 2003 [153] Rittenhouse *et al.*, 2002 [79] Nam *et al.*, 2014 [87]
Fatigue, low energy	Decreased activity in a home cage*, OFT	Pare, 1992; 1994 [26, 35] Burke *et al.*, 2016 [230]
Sense of worthlessness	NONE	-
Impaired decision making, thinking	Time in center of EPM; Spatial memory	Nosek *et al.*, 2008 [238] Grauer & Kapon, 1993 [239] Baum *et al.*, 2006 [36] Sontag *et al.*, 2013 [240]
Suicidal ideation	NONE	-

**Note:** *sex difference.

## LIST OF ABBREVIATIONS

CORT: Corticosterone
CRF: Corticotropin Releasing Factor
DB: Defensive Burying
EPM: Elevated Plus Maze
FST: Forced Swim Test
GRs: Glucocorticoid Receptors
HPA: Hypothalamic-pituitary-adrenal
MDD: Major Depressive Disorder
OFT: Open Field Test
QTL: Quantitative Trait Locus
SD: Sprague Dawley
SHR: Spontaneously Hypertensive Rat
SPT: Sucrose Preference Test
TRH: Thyrotropin Releasing Hormone
TST: Tail Suspension Test
WLI: Wistar Less Immobile
WMI: Wistar More Immobile
