# Supplementary material for: The Wistar Kyoto Rat: A Model of Depression Traits
Source: Curr Neuropharmacol. 2023 Jul 10;21(9):1884–905. doi: 10.2174/1570159X21666221129120902 (PMC10514523; doi:10.2174/1570159X21666221129120902)
Supplement: Supplementary file 1 [file CN-21-1884_SD1.pdf]

Supplementary Material

The Wistar Kyoto Rat: A Model of Depression Traits

Eva E. Redei[1,*], Mallory E. Udell[2], Leah C. Solberg-Woods[3] and Hao Chen[2]

[1]Department of Psychiatry and Behavioral Sciences, Feinberg School of Medicine, Northwestern University, Chicago, IL, USA; [2]Department of Pharmacology, Addiction Science, and Toxicology, University of Tennessee Health Science Center, Memphis, TN, USA; [3]Section on Molecular Medicine, Department of Internal Medicine, Wake Forest School of Medicine, Winston-Salem, NC, USA

1875-6190/23 $65.00+.00 **© 2023 Bentham Science Publishers**

wkyn_high_impact_final

Suppl. Table 1. High impact variant from WKY/N rats.

Whole genome sequencing data from Ramdas et.al (2019) were mapped to mRatBN7.2 reference genome. High impact variants annotated using SNPEff are shown blow.

SNP	genotype	qual	impact	gene
chr1_1654078_C_A	1/1	58	stop_gained	LOC120097698
chr1_1662667_A_C	1/1	37	stop_lost&splice_region_variant	Raet1e
chr1_5571095_C_CT	1/1	50	frameshift_variant	Shprh
chr1_7515807_AT_A	1/1	42	splice_donor_variant&intron_variant	Plagl1
chr1_7516448_AT_A	1/1	50	splice_acceptor_variant&intron_variant	Plagl1
chr1_8052656_A_G	1/1	61	stop_lost	Aig1
chr1_12088592_A_G	1/1	61	splice_donor_variant&intron_variant	LOC102546491
chr1_12171971_C_G	1/1	62	splice_acceptor_variant&intron_variant	LOC102546831
chr1_16764908_A_AG	1/1	67	splice_acceptor_variant&splice_donor_variant&intron_variant	Ptprk
chr1_16780119_AC_A	1/1	48	splice_acceptor_variant&splice_donor_variant&intron_variant	Ptprk
chr1_18990161_GA_G;chr1_18990161_G_GA	1/1	31	frameshift_variant	LOC120097350
chr1_19007680_G_A	1/1	61	splice_donor_variant&intron_variant	L3mbtl3
chr1_20502151_G_A;chr1_20502151_G_GT	1/1	57	frameshift_variant	Med23
chr1_23761715_T_TA	1/1	53	frameshift_variant	Clvs2
chr1_23761717_A_AAGATACAAT	1/1	47	stop_gained&disruptive_inframe_insertion	Clvs2
chr1_23761720_A_AATTTAAT;chr1_237617	1/1	54	frameshift_variant	Clvs2
chr1_46607593_AC_A	1/1	46	frameshift_variant	Synj2
chr1_49214695_A_AC	1/1	55	splice_donor_variant&intron_variant	LOC120100100
chr1_52632016_T_TA	1/1	32	frameshift_variant&start_lost	Rps6ka2
chr1_53017834_C_G	1/1	54	splice_donor_variant&intron_variant	Unc93a
chr1_54876495_CA_C;chr1_54876495_C_CAA	1/1	31	frameshift_variant	Vom2r9
chr1_56661911_CT_C	1/1	53	frameshift_variant	LOC108349526
chr1_60065165_AC_A	1/1	54	frameshift_variant	Vom1r10
chr1_60661837_A_T	1/1	50	stop_gained	Vom1r16
chr1_61235370_T_A	1/1	55	stop_lost	LOC103691005
chr1_61567717_A_T	0/1	30	stop_gained	Vom1r22
chr1_61790114_T_TTC	1/1	34	frameshift_variant	LOC120100076
chr1_61790583_G_GTGTT	1/1	45	frameshift_variant	LOC120100076
chr1_63240893_G_A	1/1	61	stop_gained	Vom2r16
chr1_63706676_A_T	1/1	44	stop_gained	Vom2r22
chr1_64763958_A_C	1/1	52	stop_lost	Vom1r63
chr1_64971819_A_G	1/1	59	splice_acceptor_variant&intron_variant	LOC120099261
chr1_65162378_T_C	1/1	57	splice_donor_variant&intron_variant	Vom2r26
chr1_65306447_T_G	0/1	47	stop_lost	RGD1562625
chr1_65306449_A_G	1/1	43	stop_lost	RGD1562625
chr1_65307389_CA_C	0/1	43	frameshift_variant	RGD1562625

wkyn_high_impact_final

chr1_65356509_A_C	1/1	40	splice_donor_variant&intron_variant	Lilrb3l
chr1_65504657_A_G	1/1	55	splice_donor_variant&intron_variant	Lilrb3a
chr1_65673173_A_G	1/1	60	splice_donor_variant&intron_variant	Tarm1
chr1_66757615_T_TC	1/1	37	frameshift_variant	Hiatl3
chr1_67364500_GC_G	1/1	41	frameshift_variant	Olr5
chr1_67382207_C_G	1/1	56	stop_gained	Olr6
chr1_69215430_CA_C	1/1	46	splice_acceptor_variant&splice_donor_variant&intron_variant	Ppp6r1
chr1_69215495_GC_G	1/1	44	frameshift_variant	Ppp6r1
chr1_69219823_CG_C	1/1	55	splice_acceptor_variant&splice_donor_variant&intron_variant	Tmem86b
chr1_71185402_G_A	1/1	54	start_lost	Vom2r31
chr1_72400938_AT_A	1/1	36	frameshift_variant	Vom1r47
chr1_73602293_T_G	1/1	60	splice_donor_variant&intron_variant	Zfp446
chr1_76945385_CCT_C	1/1	47	frameshift_variant	C5ar2
chr1_78028518_G_GA	1/1	40	frameshift_variant	LOC120098290
chr1_78033225_C_T	1/1	38	stop_gained	LOC120098290
chr1_78033405_T_C	1/1	60	stop_lost	LOC120098290
chr1_78511069_A_G	1/1	48	stop_lost	Micb
chr1_78898354_CA_C	1/1	57	frameshift_variant	Opa3
chr1_79046442_G_T	1/1	30	stop_gained	Klc3
chr1_80300204_T_C	1/1	62	splice_acceptor_variant&intron_variant	LOC120096890
chr1_81796565_C_CAGG	1/1	56	splice_acceptor_variant&intron_variant	LOC103691092
chr1_85230579_T_G	1/1	66	stop_gained	Zfp569
chr1_85772703_G_GC	1/1	46	frameshift_variant	RGD1560986
chr1_85816345_TA_T	1/1	58	frameshift_variant&start_lost&splice_region_variant	U2af1l4
chr1_85913653_G_A	1/1	60	splice_donor_variant&intron_variant	Haus5
chr1_86871048_A_AAG	1/1	31	frameshift_variant	Garre1
chr1_88071368_C_G	1/1	59	splice_acceptor_variant&intron_variant	Cep89
chr1_88265294_T_C	1/1	63	start_lost	Ankrd27
chr1_92752948_C_CT	1/1	35	frameshift_variant	LOC499136
chr1_92855261_TGGGTA_T	1/1	54	frameshift_variant	LOC690299
chr1_99982907_TG_T	1/1	34	frameshift_variant	LOC102557182
chr1_102666585_G_T	1/1	57	stop_gained	LOC102552387
chr1_106723335_A_T	1/1	66	splice_acceptor_variant&intron_variant	Cyfip1
chr1_108361458_G_A	1/1	63	stop_gained&splice_region_variant	Gabra5
chr1_110301660_G_A	1/1	61	stop_gained	LOC102551177
chr1_110301684_G_A	1/1	57	stop_gained	LOC102551177
chr1_110305356_C_CA	1/1	45	frameshift_variant	LOC102551177
chr1_111433875_CT_C	1/1	44	frameshift_variant	RGD1561206
chr1_119919566_TTG_T	1/1	32	splice_donor_variant&splice_region_variant&intron_variant	Lrrk1
chr1_119919569_C_G	1/1	32	splice_donor_variant&intron_variant	Lrrk1

wkyn_high_impact_final

chr1_121248266_CG_C	1/1	56	splice_donor_variant&intron_variant	Ttc23
chr1_121841596_A_G	1/1	42	stop_lost	Pgpep1l
chr1_121918119_AC_A;chr1_121918119_A_A(	1/1	56	frameshift_variant	Fam169b
chr1_122442569_CATGTATGT_C;chr1_12244	1/1	34	splice_donor_variant&splice_region_variant&intron_variant&non_codin(	LOC120099848
chr1_122466081_G_A	1/1	54	splice_donor_variant&intron_variant	LOC120099848
chr1_124344857_C_CA	1/1	60	frameshift_variant&start_lost	LOC108349002
chr1_130158426_A_T	1/1	62	stop_lost	LOC108349814
chr1_135406992_G_GA	1/1	52	splice_acceptor_variant&splice_donor_variant&intron_variant	Cpeb1
chr1_135416288_G_GC	1/1	56	splice_acceptor_variant&splice_donor_variant&intron_variant	Ap3b2
chr1_135423550_G_T;chr1_135423550_G_GT	1/1	50	frameshift_variant	Ap3b2
chr1_139815638_G_GA;chr1_139815638_G_G	1/1	50	frameshift_variant	Vom2r43
chr1_139960936_A_G	1/1	60	stop_lost&splice_region_variant	Olr16
chr1_140276688_T_TA	1/1	56	frameshift_variant	Olr27
chr1_140496463_A_T	1/1	63	start_lost	Folh1
chr1_153154758_G_A	1/1	49	stop_gained	RGD1561870
chr1_153188844_TG_T	1/1	33	frameshift_variant	Uvrag
chr1_153902815_A_T	1/1	49	splice_acceptor_variant&intron_variant	Arrb1
chr1_156472662_G_T	1/1	57	stop_gained	Trpc2
chr1_157415257_C_CA	1/1	51	frameshift_variant	Olr68
chr1_157626873_AC_A	1/1	55	frameshift_variant	Olr85
chr1_158389211_G_A	1/1	59	stop_gained	Olr135
chr1_158389686_T_TCCAGA	1/1	47	frameshift_variant	Olr135
chr1_159043000_TG_T	1/1	46	frameshift_variant	Olr165
chr1_159514691_TG_T	1/1	53	frameshift_variant	Olr193
chr1_161693999_C_CTA	1/1	55	frameshift_variant	Ovch2
chr1_161814089_AC_A	1/1	56	frameshift_variant	Olr241
chr1_163639092_A_C	1/1	57	splice_donor_variant&intron_variant	Dennd2b
chr1_164914511_GGGTTCTGAACCATGGAG	1/1	37	splice_acceptor_variant&splice_donor_variant&splice_region_variant&i	LOC120097355
chr1_168725847_T_C	1/1	63	start_lost	Pde3b
chr1_173032044_T_TG	1/1	54	splice_acceptor_variant&splice_donor_variant&intron_variant	Gde1
chr1_173032230_AC_A	1/1	44	splice_acceptor_variant&splice_donor_variant&intron_variant	Gde1
chr1_173032248_CG_C	1/1	53	splice_acceptor_variant&splice_donor_variant&intron_variant	Gde1
chr1_173032258_GA_G	1/1	45	frameshift_variant&splice_region_variant	Gde1
chr1_173890533_C_CT	1/1	46	splice_acceptor_variant&intron_variant	Acsm5
chr1_173973932_A_G	1/1	44	stop_lost	RGD1559600
chr1_173987440_C_A	1/1	61	start_lost	RGD1559600
chr1_174147066_T_TC;chr1_174147066_T_C	1/1	43	splice_donor_variant&intron_variant	Eri2
chr1_174161337_C_T	1/1	65	splice_acceptor_variant&intron_variant	Eri2
chr1_175305214_TA_T	1/1	57	frameshift_variant	Vwa3a
chr1_175340845_T_TG	1/1	55	frameshift_variant	Vwa3a

wkyn_high_impact_final

chr1_176768650_TG_T	1/1	52	splice_donor_variant&intron_variant	Chp2
chr1_176768873_C_CA	1/1	54	frameshift_variant	Chp2
chr1_180061438_G_A	1/1	58	splice_donor_variant&intron_variant	LOC103691233
chr1_181171568_G_GAT	1/1	48	frameshift_variant	Apobr
chr1_181171569_GCA_G	1/1	51	frameshift_variant	Apobr
chr1_181630729_C_T;chr1_181630729_C_CT	1/1	54	frameshift_variant	Maz
chr1_189954308_C_CG	1/1	47	frameshift_variant&splice_region_variant	Dock1
chr1_189954312_A_AT;chr1_189954312_A_T	1/1	46	frameshift_variant	Dock1
chr1_194781360_T_G	1/1	64	splice_acceptor_variant&intron_variant	Adam8
chr1_195611118_CTCACAGTCATG_C	1/1	38	frameshift_variant	Olr308
chr1_195611540_G_GT	1/1	45	frameshift_variant	Olr308
chr1_196221616_GAA_G;chr1_196221615_C	1/1	32	frameshift_variant	Ano9
chr1_196221620_GA_G;chr1_196221621_A_G	1/1	37	frameshift_variant	Ano9
chr1_196221623_C_T	1/1	43	start_lost	Ano9
chr1_196507450_T_TGA	1/1	60	frameshift_variant	Gatd1
chr1_196625722_T_C	1/1	55	splice_acceptor_variant&intron_variant	Chid1
chr1_196813334_G_GTA;chr1_196813334_G	1/1	44	frameshift_variant&splice_region_variant	Muc2
chr1_196813335_C_CCACATTT;chr1_1968133	1/1	48	frameshift_variant&splice_region_variant	Muc2
chr1_196895135_C_T	1/1	55	stop_gained	Muc5ac
chr1_198105171_T_C	1/1	63	splice_donor_variant&intron_variant	LOC120099959
chr1_198203715_T_C	1/1	60	stop_lost	Tspan32
chr1_198692718_G_A	1/1	42	splice_donor_variant&intron_variant	Slc22a18
chr1_199561302_GGT_G	1/1	56	splice_donor_variant&intron_variant	Shank2
chr1_200821057_GC_G	1/1	56	splice_acceptor_variant&splice_donor_variant&intron_variant	Lrp5
chr1_203277475_A_AT	1/1	57	frameshift_variant&splice_region_variant	Capn1
chr1_203277479_G_GA;chr1_203277479_G_A	1/1	53	frameshift_variant&stop_lost	Capn1
chr1_203277748_G_GA	1/1	35	frameshift_variant&splice_region_variant	Capn1
chr1_203277750_C_CA;chr1_203277750_C_A	1/1	34	frameshift_variant&splice_region_variant	Capn1
chr1_218216899_A_C	0/1	40	splice_acceptor_variant&intron_variant	Aldh1a7
chr1_218221999_CA_C;chr1_218221999_C_C	0/1	45	frameshift_variant	Aldh1a7
chr1_218228037_AT_A;chr1_218228037_A_AT	0/1	33	frameshift_variant	Aldh1a7
chr1_218823565_A_G	1/1	36	splice_donor_variant&intron_variant	LOC103691306
chr1_221678215_TCCTACAAGGTAACAGAAC	1/1	39	splice_acceptor_variant&splice_region_variant&intron_variant&non_co	LOC120100004
chr1_229212660_T_A	1/1	57	splice_donor_variant&intron_variant	LOC120097462
chr1_234164492_CT_C	1/1	49	frameshift_variant	Pcgf5
chr1_234507179_T_C	1/1	63	splice_donor_variant&intron_variant	LOC102553702
chr1_239566450_AG_A	1/1	51	frameshift_variant	Cc2d2b
chr1_239895087_AG_A	1/1	56	frameshift_variant	Opalin
chr1_246111099_G_A	1/1	57	stop_gained	Neurl1
chr1_246122617_AC_A;chr1_246122617_A_A	1/1	56	frameshift_variant	Neurl1

wkyn_high_impact_final

chr1_246631675_CA_C;chr1_246631674_TCA	1/1	65	frameshift_variant	Cfap43
chr1_251307088_A_AT	1/1	50	frameshift_variant	Olr385
chr1_251307497_GACTA_G	1/1	53	frameshift_variant	Olr385
chr1_252683863_T_C	1/1	63	start_lost	Rbm20
chr1_255515125_G_A	1/1	45	splice_donor_variant&intron_variant	Plekhs1
chr1_257082424_A_G	1/1	60	splice_acceptor_variant&intron_variant	Atrnl1
chr1_258626355_C_T	1/1	60	stop_gained	LOC103691392
chr1_259608143_C_T	1/1	49	start_lost	Prlhr
chr2_62227_GC_G	0/1	48	frameshift_variant	LOC120101061
chr2_1491764_GT_G	1/1	55	frameshift_variant	LOC120100635
chr2_5190406_GGGCCACTGGATCGAGGAT	1/1	31	frameshift_variant&start_lost	Ell2
chr2_5382033_CA_C	1/1	51	frameshift_variant&stop_lost	Rhobtb3
chr2_13802983_A_AT;chr2_13802983_A_ATC	1/1	51	splice_donor_variant&intron_variant	LOC103691411
chr2_13802984_C_CT;chr2_13802984_C_T	1/1	56	splice_donor_variant&intron_variant	LOC103691411
chr2_25701426_GA_G	1/1	49	frameshift_variant	Ap3b1
chr2_27848278_T_C	1/1	51	splice_acceptor_variant&intron_variant	Polk
chr2_39720365_T_C	1/1	70	start_lost	RGD1560076
chr2_39720565_C_T	1/1	63	stop_gained	RGD1560076
chr2_39720606_C_CCAAGA	1/1	32	frameshift_variant	RGD1560076
chr2_54111027_AG_A	1/1	63	frameshift_variant	C7
chr2_57208314_A_T	1/1	52	stop_lost	Nup155
chr2_57213956_GC_G	1/1	54	frameshift_variant	Nup155
chr2_57222622_GC_G	1/1	50	splice_acceptor_variant&splice_donor_variant&intron_variant	Nup155
chr2_58350008_CA_C;chr2_58350009_A_AG	1/1	42	frameshift_variant	Ugt3a2
chr2_60280866_G_A	1/1	62	stop_gained	Adamts12
chr2_65606679_A_G	1/1	64	splice_donor_variant&intron_variant	LOC120100742
chr2_65896036_A_ATGGG;chr2_65896036_	1/1	47	frameshift_variant	RGD1564125
chr2_66361857_A_C;chr2_66361857_A_AC	1/1	48	frameshift_variant	Cdh9
chr2_74810363_TATGTGCACAGGTAAAAAAC	1/1	44	splice_donor_variant&splice_region_variant&intron_variant&non_codin	LOC108350231
chr2_85263025_G_C	1/1	36	splice_acceptor_variant&intron_variant	Sirpb2
chr2_86029903_A_T	1/1	41	stop_gained	LOC102546663
chr2_87054208_C_T	0/1	65	start_lost	Lrrcc1
chr2_87058024_C_T	0/1	46	splice_acceptor_variant&intron_variant	Lrrcc1
chr2_87058025_T_C	0/1	44	splice_acceptor_variant&intron_variant	Lrrcc1
chr2_102994660_C_T	1/1	57	splice_donor_variant&intron_variant	LOC103690087
chr2_107520631_A_G;chr2_107520631_A_AG	1/1	44	frameshift_variant	LOC499584
chr2_112597625_AG_A	1/1	49	frameshift_variant	Sec62
chr2_117501217_TG_T	1/1	35	frameshift_variant	LOC685894
chr2_131071456_C_T	1/1	39	splice_donor_variant&intron_variant	RGD1563562
chr2_131637828_T_G	1/1	54	splice_donor_variant&intron_variant	LOC102555991

wkyn_high_impact_final

chr2_133559624_A_G	1/1	39	splice_acceptor_variant&intron_variant	LOC120100815
chr2_135570587_T_TGTCA	1/1	38	frameshift_variant	LOC102551737
chr2_135605608_C_T	1/1	58	stop_gained	LOC103691564
chr2_135606712_G_C	1/1	53	splice_donor_variant&intron_variant	LOC103691564
chr2_148710117_G_C	1/1	55	stop_gained	LOC120101015
chr2_148753735_A_AC	1/1	43	frameshift_variant	LOC120100835
chr2_148753738_G_GTTGA;chr2_148753738_	1/1	52	frameshift_variant	LOC120100835
chr2_148753739_C_CT;chr2_148753739_C_T	1/1	43	frameshift_variant	LOC120100835
chr2_148753825_T_A	1/1	60	stop_lost	LOC120100835
chr2_148773012_ATGGTT_A	1/1	44	frameshift_variant	LOC120100835
chr2_150034722_T_G	1/1	60	stop_lost	Lekr1
chr2_161951956_C_T	1/1	36	splice_donor_variant&intron_variant	LOC108350038
chr2_161955556_C_T	1/1	68	splice_donor_variant&intron_variant	LOC108350038
chr2_161956334_C_T	1/1	67	splice_acceptor_variant&intron_variant	LOC108350038
chr2_163926813_T_A	1/1	58	stop_gained	LOC103690241
chr2_164240103_GA_G	1/1	47	splice_acceptor_variant&splice_donor_variant&intron_variant	Rapgef2
chr2_164762289_C_T	1/1	60	splice_acceptor_variant&intron_variant	Etfdh
chr2_169577219_T_C	1/1	47	splice_donor_variant&intron_variant	LOC102550635
chr2_173530996_C_T	1/1	52	splice_acceptor_variant&intron_variant	Ttc24
chr2_174750986_T_G	1/1	51	splice_donor_variant&intron_variant	LOC120100863
chr2_176017185_AGGTG_A	1/1	39	splice_donor_variant&splice_region_variant&5_prime_UTR_variant&int	S100a16
chr2_178233114_G_A;chr2_178233110_ACTT	1/1	47	frameshift_variant	LOC120100586
chr2_178799210_G_T	1/1	45	stop_gained	Flg2
chr2_185197312_T_C	1/1	63	start_lost	Chd1l
chr2_185313646_C_T	1/1	56	splice_acceptor_variant&intron_variant	Pde4dip
chr2_186400901_G_A	1/1	56	stop_gained	LOC120100604
chr2_190611231_CAGGAAGGTGAGCGTGTC	1/1	35	splice_donor_variant&conservative_inframe_deletion&splice_region_va	Ampd1
chr2_191102804_C_CG	1/1	58	frameshift_variant	Syt6
chr2_191102813_C_T	1/1	56	stop_gained	Syt6
chr2_191321746_T_C	1/1	31	start_lost	Ap4b1
chr2_192287118_CG_C	1/1	45	frameshift_variant	LOC120101048
chr2_192937583_T_TCCTCGGGAGAGGGA	1/1	33	frameshift_variant	LOC120100907
chr2_206098835_C_A	1/1	55	splice_acceptor_variant&intron_variant	LOC120100928
chr2_210784964_AG_A	1/1	49	frameshift_variant	RGD1560826
chr2_210785116_AC_A	1/1	44	frameshift_variant	RGD1560826
chr2_210785586_TGA_T	1/1	44	frameshift_variant	RGD1560826
chr2_216007566_C_CT	1/1	54	splice_acceptor_variant&intron_variant	Larp7
chr2_218354077_C_A	1/1	66	stop_gained	Lrit3
chr2_218577301_CT_C	1/1	55	splice_acceptor_variant&intron_variant	Sec24b
chr2_223791151_C_CT	1/1	35	frameshift_variant	Slc9b1

wkyn_high_impact_final

chr2_223792580_CA_C	1/1	59	splice_acceptor_variant&splice_donor_variant&intron_variant	Slc9b1
chr2_223853727_AG_A	1/1	47	frameshift_variant&splice_region_variant	Cisd2
chr2_231310865_C_T	1/1	62	splice_donor_variant&intron_variant	Gbp4
chr2_231431952_G_T	1/1	56	stop_gained	Gbp1
chr2_231486310_G_A	1/1	60	stop_gained	Gbp3
chr2_233500128_G_C	1/1	57	splice_acceptor_variant&intron_variant	LOC102549483
chr2_243781555_G_A	1/1	63	splice_donor_variant&intron_variant	Lrrc53
chr2_244109738_T_C	1/1	64	stop_lost	Fpgt
chr3_8034097_CAGGT_C	1/1	56	splice_donor_variant&5_prime_UTR_variant&intron_variant	Fam166a
chr3_8064207_C_A	1/1	66	stop_gained&splice_region_variant	Ndor1
chr3_8541085_GGTCCTTCCCT_G	1/1	47	frameshift_variant&splice_acceptor_variant&splice_region_variant&intr	Lcn3
chr3_10140255_GA_G	1/1	56	frameshift_variant	LOC120101431
chr3_16062930_AG_A	1/1	52	frameshift_variant	Ttc16
chr3_18405781_G_A	1/1	54	stop_gained	RGD1308742
chr3_19248558_G_C	1/1	55	splice_donor_variant&intron_variant	LOC120101534
chr3_19251637_A_G	1/1	40	splice_acceptor_variant&intron_variant	LOC120101534
chr3_19419166_A_ATGAGTAGCTC	1/1	57	frameshift_variant&stop_gained	Morn5
chr3_19872134_A_AT	1/1	47	frameshift_variant	Olr399
chr3_20145506_TC_T	1/1	51	frameshift_variant	Olr404
chr3_22214760_G_A	1/1	48	splice_donor_variant&intron_variant	LOC108350366
chr3_22728911_ACCATCAGCAGAGCG_A	1/1	36	frameshift_variant	LOC102554600
chr3_27816014_G_C	1/1	64	splice_acceptor_variant&intron_variant	Kynu
chr3_35148531_AGAGTGAGAGG_A	1/1	42	frameshift_variant&splice_donor_variant&splice_region_variant&intron_	LOC499796
chr3_36395804_G_GT	1/1	51	splice_acceptor_variant&splice_donor_variant&intron_variant	Rbm43
chr3_53567640_AT_A	1/1	47	frameshift_variant	RGD1564400
chr3_65118884_G_T	1/1	59	splice_acceptor_variant&intron_variant	LOC103691836
chr3_69345861_A_G	1/1	60	splice_acceptor_variant&intron_variant	LOC120101447
chr3_71450987_T_TG	1/1	61	frameshift_variant	Olr500
chr3_71899326_A_T	1/1	67	stop_lost	Olr520
chr3_71997704_G_C	0/1	55	stop_gained	Olr528
chr3_72362554_TG_T	1/1	51	frameshift_variant	Olr545
chr3_75752726_A_AT	1/1	38	frameshift_variant	Olr715
chr3_75947276_CT_C	1/1	54	frameshift_variant	Olr724
chr3_76019594_CA_C;chr3_76019594_CAA_C	1/1	51	frameshift_variant	Olr727
chr3_77416246_GTC_G	1/1	41	frameshift_variant	RGD1309540
chr3_99655278_AG_A	1/1	38	frameshift_variant&splice_region_variant	Ryr3
chr3_99655281_AG_A	1/1	37	splice_acceptor_variant&splice_donor_variant&intron_variant	Ryr3
chr3_107971781_A_ATTTTTTTTTCT	1/1	31	frameshift_variant	Ccndbp1
chr3_108048669_G_GT	1/1	49	frameshift_variant	LOC120093166
chr3_109591267_G_T	0/1	55	splice_acceptor_variant&intron_variant	LOC120101644

wkyn_high_impact_final

chr3_109591304_GT_G	0/1	55	frameshift_variant	LOC120101644
chr3_109846791_T_A	1/1	64	splice_donor_variant&intron_variant	Sqor
chr3_112839073_GC_G	1/1	46	frameshift_variant	LOC120101650
chr3_114731328_C_A	1/1	55	stop_gained	Kcnip3
chr3_123600499_C_T	1/1	63	splice_donor_variant&intron_variant	Pak5
chr3_124357108_A_G	1/1	63	start_lost	Slx4ip
chr3_125754349_T_C	1/1	65	splice_donor_variant&intron_variant	LOC120101704
chr3_131086122_GACATGCACACTAGTGCA	1/1	45	splice_donor_variant&splice_region_variant&intron_variant&non_codin	LOC120101720
chr3_133809569_A_G	1/1	61	splice_donor_variant&intron_variant	Ralgapa2
chr3_133828287_A_G	1/1	60	start_lost	Ralgapa2
chr3_140993705_G_A	1/1	65	splice_acceptor_variant&intron_variant	Defb28
chr3_141543877_CAG_C	1/1	59	frameshift_variant	Ccm2l
chr3_144254762_CTG_C	1/1	40	frameshift_variant	Procr
chr3_144254765_CT_C;chr3_144254766_T_C	1/1	39	frameshift_variant	Procr
chr3_147032006_ACT_A	1/1	37	frameshift_variant	Snhg11
chr3_154022101_C_G	1/1	31	splice_donor_variant&intron_variant	Slc35c2
chr3_165098609_TC_T	1/1	56	frameshift_variant	LOC102551000
chr3_165390676_T_C	1/1	57	splice_donor_variant&intron_variant	LOC120101827
chr3_165580833_G_T	1/1	60	splice_acceptor_variant&intron_variant	LOC102555003
chr4_113243_AC_A	1/1	37	frameshift_variant	LOC108352017
chr4_113247_TAACCATTCTTGAAAGGC_T	1/1	33	frameshift_variant	LOC108352017
chr4_113962_A_C	1/1	44	stop_lost	LOC108352017
chr4_9655439_A_AC	1/1	58	frameshift_variant&start_lost	Kmt2c
chr4_9949694_A_ATCACC	1/1	47	frameshift_variant&stop_gained	LOC120102456
chr4_13116250_C_T	1/1	54	stop_gained	LOC103692036
chr4_20713784_G_T	1/1	63	stop_gained	LOC120102459
chr4_20714597_G_T	1/1	55	stop_gained	LOC120102459
chr4_20714817_G_GAGCCCAAGCA	1/1	46	frameshift_variant	LOC120102459
chr4_21053353_C_T	1/1	50	stop_gained	LOC102552749
chr4_21053488_T_C	1/1	55	stop_lost	LOC102552749
chr4_40119558_C_CTAGTT	1/1	43	splice_acceptor_variant&intron_variant	LOC120102235
chr4_43957424_AGGTGGAG_A	1/1	32	frameshift_variant	Rbmxl1b
chr4_55488604_C_T	1/1	68	splice_donor_variant&intron_variant	LOC120102158
chr4_59715660_T_C	1/1	64	splice_acceptor_variant&intron_variant	LOC120102485
chr4_62100257_A_T	1/1	59	stop_gained	LOC120102446
chr4_63512744_G_GT;chr4_63512744_G_GTT	1/1	36	frameshift_variant&splice_region_variant	Agbl3
chr4_68049843_T_C	1/1	31	start_lost	Chmp4bl1
chr4_69713138_C_G	1/1	57	stop_gained	Mgam
chr4_70215995_GGAAAAGCAACTTGAGTTAA	1/1	36	splice_acceptor_variant&disruptive_inframe_deletion&splice_region_va	Try5
chr4_71594728_T_C	1/1	61	splice_donor_variant&intron_variant	Tcaf1

wkyn_high_impact_final

chr4_72083276_T_C	1/1	60	splice_acceptor_variant&intron_variant	LOC102548553
chr4_77642828_GAT_G	1/1	56	frameshift_variant	Gimap4
chr4_77700179_G_A;chr4_77700179_GC_G;c	1/1	39	frameshift_variant	Gimap5
chr4_80270304_AAATCCGAG_A	1/1	49	frameshift_variant	LOC681658
chr4_80603780_AC_A	1/1	54	frameshift_variant	RGD1561341
chr4_80624812_A_T	1/1	56	stop_gained	LOC120102271
chr4_80909924_C_CAA	1/1	55	frameshift_variant	LOC120102517
chr4_81280171_C_CGGA	1/1	35	splice_donor_variant&intron_variant	LOC120102276
chr4_81682572_CTGCACGTAAGT_C	1/1	52	frameshift_variant&splice_donor_variant&splice_region_variant&intron_	LOC685406
chr4_81843385_G_GTTTGT	1/1	52	splice_donor_variant&intron_variant	Tax1bp1
chr4_84321057_T_G;chr4_84321057_T_TC	1/1	61	frameshift_variant	Inmt
chr4_90273714_GT_G;chr4_90273703_	1/1	33	splice_donor_variant&5_prime_UTR_truncation&exon_loss_variant&sp	Ccser1
chr4_91308687_T_C;chr4_91308687_T_TC	1/1	55	frameshift_variant	LOC108350839
chr4_96416574_ATTGAATTCTTACTCTCTAC	1/1	31	splice_acceptor_variant&splice_region_variant&intron_variant&non_co	LOC120102291
chr4_100775310_T_G	1/1	33	splice_donor_variant&intron_variant	LOC120102301
chr4_100776459_G_C	1/1	56	splice_acceptor_variant&intron_variant	LOC120102301
chr4_100778221_G_A	0/1	43	splice_donor_variant&intron_variant	LOC120102301
chr4_122169316_T_C	1/1	54	stop_lost	LOC102547820
chr4_125518676_G_T	1/1	50	splice_donor_variant&intron_variant	LOC102555978
chr4_126999559_ACAGTTTCCTGTGCT_A	1/1	42	splice_acceptor_variant&splice_region_variant&intron_variant&non_co	LOC120102171
chr4_144612148_T_G;chr4_144612148_T_TT	1/1	60	frameshift_variant	Grm7
chr4_146338641_G_GA	1/1	33	frameshift_variant	Lhfpl4
chr4_146338647_AAG_A	1/1	37	frameshift_variant&splice_region_variant	Lhfpl4
chr4_146338941_TC_T	1/1	56	frameshift_variant	Lhfpl4
chr4_146636414_A_G	1/1	50	splice_acceptor_variant&intron_variant	Creld1
chr4_147700867_A_C	1/1	58	stop_gained	LOC120102367
chr4_149778712_CA_C	1/1	30	frameshift_variant	Olr829
chr4_152233264_GT_G	1/1	59	splice_acceptor_variant&splice_donor_variant&intron_variant	Cacna1c
chr4_152408686_G_C	1/1	57	start_lost	Cacna2d4
chr4_153688835_A_G;chr4_153688835_A_C;c	1/1	61	frameshift_variant&synonymous_variant	Il17ra
chr4_156687093_C_T;chr4_156687092_GC_G	1/1	61	frameshift_variant	Vom2r48
chr4_161029405_T_G	1/1	50	splice_acceptor_variant&intron_variant	LOC102556609
chr4_161878598_C_T	1/1	55	splice_donor_variant&intron_variant	LOC102554736
chr4_162100266_G_A	1/1	56	stop_gained	LOC689757
chr4_162471345_T_A	1/1	55	stop_gained	Klrb1
chr4_162514252_CTT_C	1/1	39	frameshift_variant	LOC102553980
chr4_162804697_G_A	1/1	45	stop_gained	Chtopl1
chr4_162804781_C_CCATA	1/1	43	frameshift_variant	Chtopl1
chr4_163299671_T_A	1/1	50	stop_lost	Klrh1
chr4_164775455_GT_G;chr4_164775455_GTT	0/1	57	frameshift_variant&splice_acceptor_variant&splice_donor_variant&splic	Ly49s7

wkyn_high_impact_final

chr4_166252701_GA_G	1/1	40	frameshift_variant	Tas2r110
chr4_166252707_A_AG;chr4_166252707_A_G	1/1	32	frameshift_variant	Tas2r110
chr4_167597525_A_G	1/1	68	splice_donor_variant&intron_variant	Dusp16
chr4_167978094_TAC_T;chr4_167978096_C_	1/1	35	frameshift_variant	LOC120102416
chr4_170085029_T_A	1/1	64	splice_acceptor_variant&intron_variant	Rerg
chr4_177916585_G_A	1/1	56	splice_donor_variant&intron_variant	LOC102554453
chr4_178173144_A_G	1/1	68	start_lost	Cfap94
chr4_178173199_A_AG	1/1	62	frameshift_variant	Cfap94
chr4_181255911_T_C;chr4_181255911_T_CA	1/1	60	frameshift_variant&synonymous_variant	Tmtc1
chr5_5465058_ATT_A	1/1	51	frameshift_variant	Rbpjl2
chr5_5466240_CAT_C	1/1	46	frameshift_variant	Rbpjl2
chr5_9074666_A_AATTT	1/1	46	frameshift_variant&stop_lost	Arfgef1
chr5_19000946_AG_A;chr5_19000946_A_AG	1/1	49	splice_acceptor_variant&intron_variant	RGD1565372
chr5_24809415_C_A	1/1	42	stop_gained	LOC103692301
chr5_26462627_A_T	1/1	57	splice_donor_variant&intron_variant	LOC120102833
chr5_38642768_A_AT	1/1	52	frameshift_variant	LOC103695246
chr5_49275880_T_TTGTC	1/1	40	frameshift_variant	Cfap206
chr5_49585283_T_C	1/1	65	splice_donor_variant&intron_variant	LOC102549876
chr5_56598142_C_CAGCAGCAGCAGCAG;ch	1/1	37	frameshift_variant	Kif24
chr5_62697810_T_C	1/1	50	splice_acceptor_variant&intron_variant	LOC102551711
chr5_84281449_AC_A	1/1	42	frameshift_variant	LOC683573
chr5_84281452_A_AGT	1/1	39	frameshift_variant	LOC683573
chr5_90985484_CA_C	1/1	57	splice_acceptor_variant&intron_variant	LOC120103065
chr5_92368419_C_T	1/1	60	stop_gained	LOC102554881
chr5_103455620_G_GT	0/1	46	frameshift_variant	LOC120103162
chr5_108343403_A_C	1/1	54	splice_acceptor_variant&intron_variant	LOC102546952
chr5_110822961_C_G	1/1	53	splice_donor_variant&intron_variant	LOC120102925
chr5_116596027_T_G	0/1	38	stop_lost	LOC685946
chr5_116596082_C_T	0/1	35	stop_gained	LOC685946
chr5_119887399_T_C	1/1	67	splice_donor_variant&intron_variant	LOC120102939
chr5_127181382_T_A	1/1	63	stop_gained	LOC100362122
chr5_129264507_A_G	1/1	58	start_lost	Tex38
chr5_130602493_T_TC	1/1	46	frameshift_variant	Btbd19
chr5_131227321_C_T	0/1	45	stop_gained	LOC103690754
chr5_131904180_AG_A;chr5_131904180_A_A	1/1	44	frameshift_variant	Szt2
chr5_132127137_C_CT	1/1	46	frameshift_variant	LOC120102963
chr5_132216844_C_CCT	1/1	61	frameshift_variant	Olr857
chr5_134578661_AGCCAGGT_A	1/1	42	frameshift_variant	LOC680700
chr5_134579182_G_GCTTGA	1/1	50	frameshift_variant	LOC680700
chr5_138867084_AGG_A	1/1	30	frameshift_variant	Clspn

wkyn_high_impact_final

chr5_138867087_AG_A;chr5_138867088_G_A	1/1	35	frameshift_variant	Clspn
chr5_138901072_G_A	1/1	36	splice_acceptor_variant&intron_variant	RGD1563072
chr5_141823195_TTCTC_T	1/1	48	frameshift_variant	Bsdc1
chr5_147148048_G_T	1/1	54	splice_acceptor_variant&intron_variant	Rsrp1
chr5_147717492_T_C	1/1	55	splice_donor_variant&intron_variant	Stpg1
chr5_148079809_G_A	1/1	51	stop_gained	LOC120103104
chr5_150815320_T_C	1/1	65	splice_acceptor_variant&intron_variant	Vwa5b1
chr5_153030067_AC_A	1/1	55	splice_donor_variant&intron_variant	Padi6
chr5_153781324_GGCGGCAGCAGCAGCA_G	1/1	45	frameshift_variant&splice_acceptor_variant&splice_region_variant&intr	Spen
chr5_158359816_C_G	1/1	48	splice_donor_variant&intron_variant	Plod1
chr5_160493357_CAG_C	1/1	41	splice_acceptor_variant&intron_variant	LOC103692514
chr5_162602581_G_GC	1/1	51	frameshift_variant	Plekhg5
chr5_162965175_T_G	1/1	62	stop_gained&splice_region_variant	LOC500594
chr5_165651391_G_C	1/1	67	start_lost	Morn1
chr6_7650586_G_GC	1/1	41	frameshift_variant	Rhoq
chr6_7650631_T_TC	1/1	46	frameshift_variant	Rhoq
chr6_7650640_G_GC	1/1	39	frameshift_variant	Rhoq
chr6_7650692_GC_G	1/1	36	frameshift_variant	Rhoq
chr6_7650727_GC_G;chr6_7650728_C_G	1/1	32	frameshift_variant	Rhoq
chr6_7650743_T_TC	1/1	53	frameshift_variant	Rhoq
chr6_7650757_GA_G	1/1	33	frameshift_variant	Rhoq
chr6_7650787_G_GC	1/1	34	frameshift_variant	Rhoq
chr6_7650804_C_A	1/1	42	stop_gained	Rhoq
chr6_7650892_T_TC	1/1	51	frameshift_variant	Rhoq
chr6_7650901_GA_G	1/1	53	frameshift_variant	Rhoq
chr6_7650913_TC_T	1/1	45	frameshift_variant	Rhoq
chr6_7650937_GGA_G	1/1	33	frameshift_variant	Rhoq
chr6_7650947_C_CA	1/1	32	frameshift_variant	Rhoq
chr6_7650967_AC_A	1/1	32	frameshift_variant	Rhoq
chr6_7650994_G_GC	1/1	33	frameshift_variant	Rhoq
chr6_7651000_G_GC	1/1	33	frameshift_variant	Rhoq
chr6_7651045_G_GC	1/1	34	frameshift_variant	Rhoq
chr6_7651057_GC_G	1/1	31	frameshift_variant	Rhoq
chr6_7652032_AC_A	1/1	48	frameshift_variant	Rhoq
chr6_9588203_CATTTGTTACCTCGTGTCAT	1/1	35	splice_donor_variant&splice_region_variant&intron_variant&non_codin	LOC103692550
chr6_10002039_C_CA	1/1	52	frameshift_variant	Dync2li1
chr6_13974648_G_GT	1/1	58	frameshift_variant	Thumpd2
chr6_23663124_CA_C	1/1	53	frameshift_variant&splice_region_variant	Clip4
chr6_23828283_C_T	1/1	59	splice_donor_variant&intron_variant	Togaram2
chr6_23828461_G_GGA	1/1	35	frameshift_variant	Togaram2

wkyn_high_impact_final

chr6_24096984_G_A	1/1	46	splice_acceptor_variant&intron_variant	LOC120103485
chr6_26505458_AGGG_A;chr6_26505458_A_A	1/1	46	frameshift_variant	Asxl2
chr6_27787333_G_C	1/1	55	splice_acceptor_variant&intron_variant	Pfn4
chr6_33961962_T_C	1/1	52	splice_donor_variant&intron_variant	LOC102547959
chr6_41194341_G_A;chr6_41194340_AG_A	1/1	61	frameshift_variant	Taf1b
chr6_41340914_G_GGCTGAGGTAGGTC	1/1	51	frameshift_variant&stop_gained	Rrm2
chr6_49751932_CTAAAGA_C	1/1	46	splice_acceptor_variant&splice_region_variant&intron_variant	LOC102547703
chr6_56504906_G_A	1/1	52	stop_gained	Smarce1l
chr6_56637022_A_AG	1/1	53	frameshift_variant	LOC120103419
chr6_66694460_T_C	1/1	63	splice_donor_variant&intron_variant	LOC102546421
chr6_72579835_A_G	1/1	42	splice_donor_variant&intron_variant	RGD1304624
chr6_83183310_A_C	1/1	65	stop_gained	Mis18bp1
chr6_92664664_G_GTATT	1/1	53	splice_donor_variant&intron_variant	Hif1a
chr6_95125611_C_T	1/1	60	stop_gained	Zbtb1
chr6_97845013_CT_C;chr6_97845013_C_CT;c	1/1	36	splice_acceptor_variant&intron_variant	Plekhh1
chr6_97848666_T_C	1/1	31	splice_donor_variant&intron_variant	Plekhh1
chr6_98082704_C_T	1/1	62	splice_acceptor_variant&intron_variant	Zfyve26
chr6_99270465_C_A	1/1	61	stop_gained	RGD1559921
chr6_100030427_C_T	1/1	41	stop_gained	LOC685190
chr6_104080567_T_TAGAGCAGCACAGCA	1/1	57	frameshift_variant	Bbof1
chr6_104249017_T_TGAAAG	1/1	42	frameshift_variant	Abcd4
chr6_105796457_TG_T	1/1	62	frameshift_variant	Ift43
chr6_105796490_A_AT;chr6_105796490_AT_A	1/1	38	frameshift_variant	Ift43
chr6_114425534_G_A	1/1	59	splice_acceptor_variant&intron_variant	LOC102549466
chr6_118981202_AAAGT_A	1/1	53	splice_donor_variant&splice_region_variant&intron_variant&non_codin	LOC108351290
chr6_123066378_G_A	1/1	56	stop_gained	Serpina3m
chr6_127280112_T_G	1/1	54	splice_acceptor_variant&intron_variant	LOC120103619
chr6_130966350_G_GA	1/1	45	frameshift_variant&stop_lost	LOC100363116
chr6_131438475_G_C	1/1	51	splice_donor_variant&intron_variant	LOC102549170
chr6_131796475_G_GTA	1/1	56	splice_donor_variant&intron_variant	Cep170b
chr6_131929907_T_G	1/1	54	splice_acceptor_variant&intron_variant	Gpr132
chr6_133784359_T_TA	1/1	39	frameshift_variant&splice_region_variant	LOC120103436
chr6_139071175_A_AT	1/1	48	frameshift_variant	Dnah11
chr7_139216_A_G	1/1	42	splice_acceptor_variant&intron_variant	LOC102546625
chr7_323059_GA_G	1/1	48	frameshift_variant	LOC120093523
chr7_687919_A_T;chr7_687919_A_AG	1/1	57	frameshift_variant	Apon
chr7_701106_T_TGCCATTTCACACAGAGA	1/1	55	frameshift_variant	Apof
chr7_710631_AC_A	1/1	37	frameshift_variant	Stat2
chr7_1388373_GC_G	1/1	43	frameshift_variant	Itga7
chr7_1388376_AC_A	1/1	49	splice_acceptor_variant&splice_donor_variant&intron_variant	Itga7

wkyn_high_impact_final

chr7_4611188_ACCCAGGAGCT_A	1/1	45	frameshift_variant	Olr987
chr7_6678680_AT_A	1/1	47	frameshift_variant	Olr1065
chr7_8125079_ACT_A	1/1	49	frameshift_variant	Tle2
chr7_8148817_A_AC;chr7_8148817_A_ACC	1/1	44	frameshift_variant	Tle5
chr7_8847780_C_CCTGT	1/1	57	frameshift_variant	Sppl2b
chr7_8904314_G_A;chr7_8904314_G_GC	1/1	55	frameshift_variant	Jsrp1
chr7_8959458_A_ATGGGTCCAGAGGGAGA	1/1	38	start_lost&conservative_inframe_insertion	Dot1l
chr7_9349758_A_G	1/1	62	splice_donor_variant&intron_variant	Plk5
chr7_9669873_G_GCTCTCT;chr7_9669873_G	1/1	36	frameshift_variant	Polr2e
chr7_11574283_A_G	1/1	54	splice_acceptor_variant&intron_variant	Cyp4f37
chr7_11587110_A_T	1/1	62	splice_acceptor_variant&intron_variant	Cyp4f37
chr7_11664962_C_T	1/1	54	stop_gained	LOC103692784
chr7_12622373_C_A	1/1	34	stop_gained	LOC120093538
chr7_12840252_A_T	1/1	39	stop_gained	Olr1095
chr7_17317288_G_T	1/1	54	splice_acceptor_variant&intron_variant	RGD1564409
chr7_28545425_A_C	1/1	65	splice_acceptor_variant&intron_variant	LOC120093556
chr7_29940575_T_A	1/1	60	splice_acceptor_variant&intron_variant	Cradd
chr7_30775314_G_GTCTGCCACCATCTA;chr	1/1	47	frameshift_variant&stop_gained	Plekhg7
chr7_46968528_T_TCG;chr7_46968528_T_G	1/1	38	frameshift_variant	Phlda1
chr7_58715939_G_A	1/1	48	stop_gained	LOC108351468
chr7_59529336_T_A	1/1	58	splice_donor_variant&intron_variant	LOC120093624
chr7_62863898_A_G	1/1	55	stop_lost	Eef1akmt3
chr7_63016327_TG_T	1/1	52	frameshift_variant	Dtx3
chr7_65763258_G_GC	1/1	34	frameshift_variant	LOC120093874
chr7_101239123_G_A	1/1	58	splice_donor_variant&intron_variant	LOC120093889
chr7_105988360_T_C	1/1	38	splice_acceptor_variant&intron_variant	LOC120093890
chr7_107011982_A_AC	1/1	56	frameshift_variant	LOC300024
chr7_108598898_T_C	1/1	62	splice_donor_variant&intron_variant	Zfp7
chr7_108621949_A_G	1/1	56	splice_donor_variant&intron_variant	Commd5
chr7_109262213_G_T	1/1	54	stop_gained	Apol11a
chr7_111783480_T_G	1/1	53	splice_acceptor_variant&intron_variant	LOC120093694
chr7_112225272_C_CTGCGTCG	1/1	49	frameshift_variant	Fam83f
chr7_113815488_A_AG	1/1	53	frameshift_variant	Wbp2nl
chr7_114261382_A_G	1/1	62	splice_acceptor_variant&intron_variant	Serhl2
chr7_118833033_C_T	1/1	63	stop_gained	Tafa5
chr7_120066616_A_AC	1/1	49	frameshift_variant	Mlc1
chr7_120430847_AGGTACAACACTGGCCAA	1/1	35	frameshift_variant&splice_donor_variant&splice_region_variant&intron_	Ncaph2
chr7_124411126_T_A	1/1	62	splice_acceptor_variant&intron_variant	Gxylt1
chr7_128577289_AC_A	1/1	62	frameshift_variant	RGD1565798
chr7_133130312_A_G	1/1	61	start_lost	Krt8

wkyn_high_impact_final

chr7_134481979_T_C	1/1	60	splice_acceptor_variant&intron_variant	Itga5
chr7_134737691_G_A	1/1	51	splice_acceptor_variant&intron_variant	LOC102547969
chr8_744919_TG_T	1/1	52	splice_acceptor_variant&splice_donor_variant&intron_variant	Gucy1a2
chr8_745282_CT_C	1/1	42	frameshift_variant	Gucy1a2
chr8_5850855_A_AG;chr8_5850855_A_AGC	1/1	43	frameshift_variant&splice_region_variant	Trpc6
chr8_5850857_C_CT;chr8_5850857_C_T	1/1	51	frameshift_variant&splice_region_variant	Trpc6
chr8_5850895_T_TG	1/1	47	frameshift_variant	Trpc6
chr8_5850898_GT_G	1/1	53	frameshift_variant	Trpc6
chr8_14309190_C_A	1/1	62	splice_donor_variant&intron_variant	LOC500948
chr8_15657348_TACAG_T	1/1	56	frameshift_variant	LOC102555809
chr8_17187243_C_T	1/1	58	stop_gained	Olr1146
chr8_17310559_CAT_C	1/1	57	frameshift_variant&stop_lost&splice_region_variant	Olr1149
chr8_17311492_T_TC	1/1	53	frameshift_variant	Olr1149
chr8_18292262_CTT_C	1/1	55	splice_acceptor_variant&splice_donor_variant&intron_variant	Olr1867
chr8_18292718_T_G	1/1	49	stop_lost	Olr1867
chr8_27933665_C_T	1/1	64	splice_acceptor_variant&intron_variant	LOC120094319
chr8_34024856_A_G	1/1	48	splice_acceptor_variant&intron_variant	Pate2
chr8_37135669_C_T	1/1	64	stop_gained	Robo3
chr8_38068725_G_GA	1/1	46	frameshift_variant	Olr1217
chr8_38320336_CT_C;chr8_38320337_T_C	1/1	54	frameshift_variant	Olr1220
chr8_38320341_G_GA;chr8_38320341_G_A	1/1	45	frameshift_variant	Olr1220
chr8_38377973_A_AT	1/1	56	frameshift_variant	LOC108351823
chr8_40358571_G_GGT	1/1	46	frameshift_variant	Olr1335
chr8_40790337_G_GT	1/1	62	frameshift_variant	Gramd1b
chr8_44451241_A_AGCCG	1/1	59	frameshift_variant	C1qtnf5
chr8_44678797_A_G	1/1	54	start_lost	Hmbs
chr8_45552338_A_AC	1/1	53	frameshift_variant	Smim35
chr8_46084693_T_TCC	1/1	33	frameshift_variant	Cep164
chr8_46566650_G_GTGAGCAGGGA	1/1	44	splice_donor_variant&intron_variant	Zpr1
chr8_48941633_T_C	1/1	60	stop_lost	Nnmt
chr8_49782951_C_CT	1/1	53	frameshift_variant	Ankk1
chr8_51112897_A_C	1/1	55	splice_donor_variant&intron_variant	RGD1564937
chr8_55316336_CTGGAGG_C	1/1	37	splice_donor_variant&splice_region_variant&5_prime_UTR_variant&int	Hykk
chr8_58532590_C_CTG	1/1	39	frameshift_variant&splice_region_variant	Ccdc33
chr8_63660574_GGGGAGAC_G	1/1	32	frameshift_variant	Map2k5
chr8_66117606_C_CAATA	1/1	49	frameshift_variant&stop_gained	Pif1
chr8_68468485_G_T	1/1	55	stop_gained	C2cd4a
chr8_76379021_AG_A;chr8_76379021_A_AG	1/1	55	frameshift_variant	Lysmd2
chr8_79417996_AC_A	1/1	51	splice_acceptor_variant&splice_donor_variant&intron_variant	Slc17a5
chr8_84528258_GA_G;chr8_84528258_G_GA	1/1	46	frameshift_variant	Sh3bgrl2

wkyn_high_impact_final

chr8_86767602_A_G;chr8_86767602_A_C	1/1	49	stop_lost	RGD1564645
chr8_86767669_C_CCTCA	1/1	52	frameshift_variant	RGD1564645
chr8_89634535_T_C	1/1	60	splice_acceptor_variant&intron_variant	RGD1560775
chr8_89634647_T_TATTAAGC	1/1	44	frameshift_variant&stop_gained	RGD1560775
chr8_89634651_G_GTT	1/1	41	frameshift_variant	RGD1560775
chr8_89713063_C_T	1/1	58	splice_acceptor_variant&intron_variant	RGD1560775
chr8_90706388_C_CT	1/1	40	frameshift_variant	Adamts7
chr8_96226749_C_CA	1/1	41	splice_acceptor_variant&splice_donor_variant&intron_variant	PCOLCE2
chr8_96548408_T_C	1/1	60	splice_donor_variant&intron_variant	Xrn1
chr8_96875655_ACTGT_A	1/1	30	frameshift_variant	LOC102550734
chr8_97419951_CT_C	1/1	60	splice_acceptor_variant&intron_variant	Pxylp1
chr8_99564070_CT_C	1/1	56	frameshift_variant	Faim
chr8_99792144_C_CG;chr8_99792144_C_CA	1/1	53	frameshift_variant	LOC684466
chr8_99825282_T_A	1/1	57	splice_acceptor_variant&intron_variant	Cep70
chr8_100030857_T_TCAGTATGGCA;chr8_100	1/1	55	frameshift_variant	Nme9
chr8_105864200_G_GATTGAGAGCCCCA	1/1	55	frameshift_variant	Nek11
chr8_106855626_GGT_G;chr8_106855626_G	1/1	39	splice_donor_variant&intron_variant	Twf2
chr8_108666111_A_G	1/1	58	start_lost	Uba7
chr8_108770485_G_A	1/1	57	splice_donor_variant&intron_variant	Mst1
chr8_109388639_TG_T	1/1	59	frameshift_variant	LOC679586
chr8_109550676_GC_G	1/1	50	frameshift_variant	Celsr3
chr8_110459621_A_G	1/1	61	start_lost	Kif9
chr8_110938273_GGACT_G	1/1	39	frameshift_variant	Tdgf1
chr8_110938312_TGGGG_T;chr8_110938312	1/1	30	frameshift_variant	Tdgf1
chr8_114024496_T_C	1/1	61	start_lost&splice_region_variant	Susd5
chr8_118241159_TGGAGAAGCTGGAGGAGG	1/1	44	splice_donor_variant&disruptive_inframe_deletion&splice_region_varia	Golga4
chr8_118260159_AG_A	1/1	47	frameshift_variant	Golga4
chr8_118260514_GT_G	1/1	54	frameshift_variant	Golga4
chr8_118260522_CT_C	1/1	31	frameshift_variant	Golga4
chr8_119112891_ACAGATACACACACACACA	1/1	37	splice_acceptor_variant&splice_donor_variant&splice_region_variant&i	Xylb
chr8_121155932_C_A	1/1	56	splice_donor_variant&intron_variant	Cck
chr8_122772987_T_A	1/1	55	splice_acceptor_variant&intron_variant	Zdhhc3
chr9_1143925_GTCATGCTTTTGTGAGTGACC	1/1	36	frameshift_variant&splice_donor_variant&splice_region_variant&intron_	Uhrf1
chr9_2184438_CTCTCTGTGTGTCATTTCTGA	1/1	42	splice_acceptor_variant&splice_region_variant&5_prime_UTR_variant&	Vav1
chr9_6962642_G_A	1/1	34	stop_gained	LOC120093086
chr9_7177706_G_A	1/1	46	stop_gained	RGD1559960
chr9_7177728_AT_A	1/1	40	frameshift_variant	RGD1559960
chr9_7177741_A_G	1/1	45	start_lost	RGD1559960
chr9_12416871_C_T	1/1	62	splice_donor_variant&intron_variant	LOC120094632
chr9_16889072_AC_A	1/1	47	frameshift_variant	Enpp4

wkyn_high_impact_final

chr9_17308009_GAA_G	1/1	41	frameshift_variant	Slc25a27
chr9_20109231_G_A	1/1	51	splice_donor_variant&intron_variant	Crisp2
chr9_20883880_G_A	1/1	54	stop_gained	Defb17
chr9_45993234_T_TGCC	1/1	47	start_lost&conservative_inframe_insertion	Ecrg4
chr9_46035696_C_T	1/1	40	splice_donor_variant&intron_variant	LOC120094680
chr9_48248185_A_G	1/1	46	stop_lost	Ormdl1
chr9_48248770_CT_C	1/1	62	frameshift_variant	Ormdl1
chr9_48248807_G_GT	1/1	54	frameshift_variant	Ormdl1
chr9_60567841_TACCTAGGAAGCACAA_T	1/1	41	splice_donor_variant&5_prime_UTR_variant&intron_variant	Tmem237
chr9_61073096_GA_G	1/1	55	frameshift_variant	RGD1562029
chr9_64982757_T_TG	1/1	53	frameshift_variant	Adam23
chr9_64982759_GT_G	1/1	46	frameshift_variant	Adam23
chr9_65903481_CG_C	1/1	45	frameshift_variant	LOC120094582
chr9_76750211_G_A	1/1	55	splice_donor_variant&intron_variant	LOC120094712
chr9_84125181_CG_C	1/1	52	frameshift_variant	Agfg1
chr9_84125189_GC_G	1/1	47	frameshift_variant&splice_region_variant	Agfg1
chr9_84125228_GCC_G	1/1	46	frameshift_variant	Agfg1
chr9_86172819_C_G	1/1	56	stop_gained	Csprs
chr9_86322216_T_TC	1/1	33	splice_acceptor_variant&intron_variant	Sp100
chr9_94279074_T_C	1/1	58	start_lost	Thap4
chr9_94398472_T_C	1/1	60	start_lost	Neu4
chr9_106018448_T_TG;chr9_106018448_T_T	1/1	46	frameshift_variant	LOC102553382
chr9_106115740_T_C	1/1	46	splice_acceptor_variant&intron_variant	LOC108348307
chr9_107271406_T_C	1/1	67	splice_donor_variant&intron_variant	Ptprm
chr9_111034222_GTTTTGTCTTCTTTCTTTTT	1/1	37	splice_acceptor_variant&splice_donor_variant&splice_region_variant&i	LOC120094779
chr9_113344515_CA_C	1/1	60	frameshift_variant	Clul1
chr9_113949172_C_T	1/1	55	stop_gained	Rpl5l1
chr10_2118987_GA_G	1/1	50	splice_acceptor_variant&splice_donor_variant&intron_variant	Ntan1
chr10_3938137_A_G;chr10_3938088_G	1/1	34	frameshift_variant&splice_acceptor_variant&splice_region_variant&intr	Snx29
chr10_4725730_T_TGTGCGGCTCA	1/1	49	frameshift_variant	LOC102552269
chr10_4730631_AGG_A	1/1	35	frameshift_variant	LOC102552269
chr10_4730643_G_GCCTCA	1/1	33	frameshift_variant	LOC102552269
chr10_4730644_G_GC;chr10_4730644_G_C	1/1	32	frameshift_variant	LOC102552269
chr10_4794275_T_TGC	1/1	38	frameshift_variant	LOC102552269
chr10_5262078_CA_C	1/1	50	splice_acceptor_variant&splice_donor_variant&intron_variant	Nubp1
chr10_6946741_T_TG	1/1	57	frameshift_variant	Carhsp1
chr10_6946918_G_GC	1/1	40	frameshift_variant	Carhsp1
chr10_10269043_CCG_C	1/1	47	frameshift_variant	Rbm25l1
chr10_12094484_G_A	1/1	60	stop_gained	Olr1365
chr10_12289662_T_TG	1/1	33	frameshift_variant	Olr1374

wkyn_high_impact_final

chr10_14339690_TC_T	1/1	40	frameshift_variant	Prss29
chr10_16520573_TCCTTATATCGG_T	1/1	41	frameshift_variant	LOC100910596
chr10_19038443_AG_A	1/1	43	frameshift_variant	Dock2
chr10_25178982_CT_C	1/1	54	splice_acceptor_variant&intron_variant	Ccng1
chr10_31194775_ACT_A	1/1	57	frameshift_variant	Timd4
chr10_33302420_A_G	1/1	40	splice_donor_variant&intron_variant	LOC102557028
chr10_33534206_A_G	1/1	61	splice_donor_variant&intron_variant	Zfp62
chr10_33556543_C_A	1/1	57	splice_acceptor_variant&intron_variant	LOC120095086
chr10_33941943_C_CT	1/1	52	splice_acceptor_variant&intron_variant	Flt4
chr10_37480280_C_CA	1/1	32	frameshift_variant	Zcchc10
chr10_40269518_A_T	1/1	32	stop_gained	LOC102552619
chr10_40281201_T_TG;chr10_40281201_T_TT	1/1	60	frameshift_variant	LOC120095104
chr10_42565303_G_A;chr10_42565302_AG_A	1/1	39	frameshift_variant	Lypd8
chr10_42565308_A_G;chr10_42565308_A_AG	1/1	45	frameshift_variant	Lypd8
chr10_43117074_C_A	1/1	49	stop_gained	Olr1436
chr10_43269830_CT_C	1/1	54	frameshift_variant	Olr1442
chr10_43562953_C_A	1/1	59	stop_gained	Olr1459
chr10_46608542_CG_C	1/1	56	frameshift_variant	Akap10
chr10_47126934_G_A	1/1	42	splice_donor_variant&intron_variant	Ncor1
chr10_51233206_G_GC	1/1	62	frameshift_variant	LOC102554448
chr10_53207435_C_T	1/1	57	splice_donor_variant&intron_variant	Pik3r6
chr10_54308742_CT_C	0/1	33	frameshift_variant	Tp53
chr10_54663887_GC_G	1/1	51	frameshift_variant	Ybx2
chr10_55067715_AG_A	1/1	46	frameshift_variant	Alox15
chr10_55068421_A_AAAAACAAAAC;chr10_5	1/1	51	frameshift_variant	Alox15
chr10_58070795_G_GAT	1/1	53	frameshift_variant&stop_gained	Olr1472
chr10_58349633_A_T	1/1	60	stop_gained	Olr1486
chr10_58446342_A_AT	1/1	49	frameshift_variant	Olr1491
chr10_58469157_G_GA	1/1	57	frameshift_variant	Olr1492
chr10_58862049_T_G	1/1	58	start_lost	LOC687893
chr10_58862568_T_TC	1/1	57	frameshift_variant	LOC687893
chr10_62772678_T_C	1/1	63	start_lost	Pipox
chr10_66314789_T_C	1/1	60	splice_donor_variant&intron_variant	LOC120095274
chr10_66906039_TCTCTCCCTCTCC_T;chr10	1/1	34	gene_fusion	Asic2&LOC12009527[illegible]
chr10_67527195_T_C	1/1	58	splice_acceptor_variant&intron_variant	LOC120095155
chr10_73757615_TCA_T	1/1	34	frameshift_variant	LOC100363835
chr10_73757623_A_AC;chr10_73757623_A_G	1/1	32	frameshift_variant	LOC100363835
chr10_75483469_ACGAATGAGAGGGTTTTTT	1/1	36	splice_donor_variant&splice_region_variant&intron_variant	Tom1l1
chr10_80274089_CCG_C	1/1	53	frameshift_variant	RGD1565395
chr10_82016755_A_AGG	1/1	57	splice_donor_variant&intron_variant	Scrn2

wkyn_high_impact_final

chr10_82020490_G_C;chr10_82020490_G_GC	1/1	60	frameshift_variant	Scrn2
chr10_82669739_GGT_G	1/1	44	splice_donor_variant&intron_variant	Mllt6
chr10_82985484_T_C	1/1	61	splice_acceptor_variant&intron_variant	Arl5c
chr10_83428203_CGTGAGTGGAGGGA_C	1/1	31	splice_donor_variant&splice_region_variant&intron_variant	Erbb2
chr10_84130769_G_C	1/1	51	splice_donor_variant&intron_variant	LOC120095183
chr10_84592301_ACAGCAGGGC_A	1/1	37	splice_acceptor_variant&disruptive_inframe_deletion&splice_region_va	LOC680160
chr10_86188742_AT_A	1/1	50	frameshift_variant	Ramp2
chr10_88084171_CG_C	0/1	49	frameshift_variant	Hexim2
chr10_88084318_CCGCAGCAAGCAGGAGCT_	0/1	52	frameshift_variant	Hexim2
chr10_88717263_AAG_A	1/1	55	frameshift_variant	Wnt3
chr10_89557356_GGCTGGTCACAGTCCCGTT	1/1	35	frameshift_variant&splice_donor_variant&splice_region_variant&intron_	Itgb3
chr10_91437895_A_G	1/1	54	stop_lost	Tex2
chr10_94038032_G_A	1/1	55	splice_acceptor_variant&intron_variant	LOC102550112
chr10_105989910_C_T	1/1	43	stop_gained	Aspscr1
chr10_105994503_CCT_C	1/1	50	frameshift_variant	Lrrc45
chr11_27448941_CAGGT_C	1/1	51	splice_donor_variant&splice_region_variant&intron_variant&non_codin	LOC108352301
chr11_28430229_A_G	1/1	56	stop_lost	LOC100359452
chr11_28442144_G_GC	1/1	39	frameshift_variant	LOC102551771
chr11_28442147_AT_A	1/1	38	frameshift_variant	LOC102551771
chr11_31383747_C_CGGGA	1/1	48	frameshift_variant	LOC103693540
chr11_31393164_A_AGTGG	1/1	48	frameshift_variant	LOC103693540
chr11_40884767_T_C	1/1	58	start_lost	Riox2
chr11_41152219_A_AGT	1/1	44	frameshift_variant	Olr1536
chr11_41152220_AC_A	1/1	45	frameshift_variant	Olr1536
chr11_41152225_GA_G;chr11_41152226_A_G	1/1	42	frameshift_variant	Olr1536
chr11_44650157_C_CA	1/1	57	splice_donor_variant&intron_variant	Zbtb11
chr11_55081443_G_A	1/1	49	stop_gained	Abhd10
chr11_55945806_GA_G;chr11_55945806_G_G	1/1	45	frameshift_variant&start_lost	Gtpbp8
chr11_61911561_C_T	1/1	36	stop_gained	RGD1563835
chr11_62002593_GCTGTTCACCTGAGT_G	1/1	43	splice_donor_variant&splice_region_variant&intron_variant&non_codin	B4galt4
chr11_62454615_A_G	1/1	31	stop_lost	Cfap91
chr11_63414842_CT_C	1/1	56	frameshift_variant	Stxbp5l
chr11_66980190_A_G	1/1	44	start_lost	Muc13
chr11_70087793_C_T	1/1	57	splice_acceptor_variant&intron_variant	LOC102549085
chr11_71475839_C_T	1/1	59	stop_gained	Atp13a5
chr11_80322341_T_G	1/1	65	splice_acceptor_variant&intron_variant	Vwa5b2
chr11_81278606_T_C	1/1	44	start_lost	Olr1566
chr11_81798560_G_A	1/1	61	splice_acceptor_variant&intron_variant	LOC680329
chr11_81798571_T_C	1/1	53	splice_donor_variant&intron_variant	LOC680329
chr11_81838927_A_G	1/1	51	start_lost	LOC680329

wkyn_high_impact_final

chr11_81910083_C_G	1/1	54	splice_acceptor_variant&intron_variant	LOC680329
chr11_81910084_C_T	1/1	50	splice_acceptor_variant&intron_variant	LOC680329
chr11_81910085_C_A	1/1	52	splice_acceptor_variant&intron_variant	LOC680329
chr11_81910087_G_A	1/1	53	splice_acceptor_variant&intron_variant	LOC680329
chr11_83003598_T_A	1/1	56	splice_acceptor_variant&intron_variant	LOC103690707
chr12_581626_C_T	1/1	61	stop_gained&splice_region_variant	Stard13
chr12_2280804_A_G	1/1	53	splice_acceptor_variant&intron_variant	LOC120095922
chr12_10212095_A_G	1/1	59	splice_acceptor_variant&intron_variant	LOC100360200
chr12_10326986_A_G	1/1	67	splice_acceptor_variant&intron_variant	Baiap2l1
chr12_12031035_CAG_C	1/1	53	frameshift_variant	Radil
chr12_17071641_GC_G	1/1	44	splice_acceptor_variant&splice_donor_variant&intron_variant	Mblac1
chr12_17071650_GC_G	1/1	41	splice_acceptor_variant&splice_donor_variant&intron_variant	Mblac1
chr12_17127744_T_TCATG	1/1	39	frameshift_variant&splice_region_variant	LOC680825
chr12_18448390_A_AT	1/1	44	frameshift_variant	Vom2r64
chr12_19498027_AGGGCT_A	1/1	45	frameshift_variant&stop_lost&splice_region_variant	LOC120095957
chr12_19508401_T_A	1/1	42	splice_donor_variant&intron_variant	LOC120095958
chr12_19530961_G_GGA	1/1	51	frameshift_variant	Muc3
chr12_19532613_GT_G	1/1	57	frameshift_variant	Muc3
chr12_19532658_GA_G	1/1	37	frameshift_variant	Muc3
chr12_19532671_T_TG	1/1	44	frameshift_variant&splice_region_variant	Muc3
chr12_19757432_CACCGTAGA_C	1/1	43	splice_donor_variant&splice_region_variant&intron_variant&non_codin	LOC100910802
chr12_26876999_C_CTG	1/1	59	frameshift_variant	Cct6a
chr12_26949109_G_A	1/1	50	stop_gained	Mrps17
chr12_28246885_T_C	1/1	61	splice_donor_variant&intron_variant	LOC120095942
chr12_28250986_A_G	1/1	60	splice_acceptor_variant&intron_variant	LOC120095942
chr12_29117084_A_G;chr12_29117084_A_AC	1/1	64	frameshift_variant	Tmem132c
chr12_31642095_GA_G	1/1	58	splice_acceptor_variant&splice_donor_variant&intron_variant	Rflna
chr12_35624625_G_T	1/1	58	start_lost	Oas1i
chr12_35626814_CT_C	1/1	51	frameshift_variant	Oas1i
chr12_36657030_A_G	1/1	64	splice_donor_variant&intron_variant	LOC120095982
chr12_41749736_T_C	1/1	58	start_lost	LOC691311
chr12_41794792_C_A	1/1	62	stop_gained	RGD1560398
chr12_41828004_GC_G	1/1	47	frameshift_variant	Ankrd13a
chr12_45829024_G_GGCTAT	1/1	55	frameshift_variant	Hscb
chr12_46576906_T_TC;chr12_46576906_T_TC	1/1	47	frameshift_variant	Gtpbp6
chr13_9364975_A_G	1/1	45	splice_donor_variant&intron_variant	LOC108352511
chr13_10007160_GGGGC_G;chr13_10007164_	1/1	30	frameshift_variant	LOC120096223
chr13_10859716_CAG_C	1/1	54	frameshift_variant	LOC100912998
chr13_19522483_C_CG;chr13_19522483_C_C	1/1	40	frameshift_variant	LOC103691625
chr13_23242588_C_A	0/1	49	splice_acceptor_variant&intron_variant	Serpinb3a

wkyn_high_impact_final

chr13_23242612_G_A;chr13_23242612_G_GT	0/1	55	frameshift_variant	Serpinb3a
chr13_23252211_C_A	1/1	40	stop_gained	LOC120096217
chr13_23252357_G_GA	1/1	57	frameshift_variant&splice_region_variant	LOC120096217
chr13_23256472_A_G	1/1	66	stop_lost&splice_region_variant	LOC120096217
chr13_23570696_C_T	1/1	59	stop_gained	Serpinb10
chr13_25622632_A_C	1/1	53	stop_lost&splice_region_variant	LOC108353429
chr13_26090333_AT_A	1/1	57	frameshift_variant	RGD1560523
chr13_28357764_T_TG	1/1	46	frameshift_variant	LOC108352638
chr13_29484457_A_T	1/1	39	stop_gained	LOC102547021
chr13_29849834_T_C	1/1	51	splice_donor_variant&intron_variant	LOC120096197
chr13_29851933_G_A	1/1	48	splice_donor_variant&intron_variant	LOC120096197
chr13_31190048_A_G	1/1	61	stop_lost	Sctr
chr13_38888004_A_G	1/1	57	stop_lost&splice_region_variant	Mgat5
chr13_40168519_C_T	1/1	49	splice_acceptor_variant&intron_variant	LOC102548838
chr13_42159790_T_A	1/1	54	splice_acceptor_variant&intron_variant	Pfkfb2
chr13_45642147_CA_C	1/1	36	frameshift_variant&start_lost	Chi3l1
chr13_71518306_GAC_G	1/1	43	frameshift_variant&splice_acceptor_variant&splice_donor_variant&splic	Cop1
chr13_71518309_A_C	1/1	54	splice_acceptor_variant&splice_donor_variant&intron_variant	Cop1
chr13_71521891_AG_A	1/1	48	frameshift_variant	Cop1
chr13_71521965_TA_T	1/1	53	splice_acceptor_variant&splice_donor_variant&intron_variant	Cop1
chr13_73691608_G_GAAAA;chr13_73691608	1/1	32	frameshift_variant	LOC108352562
chr13_73830634_C_T	1/1	55	splice_donor_variant&intron_variant	LOC103692000
chr13_75226006_A_G	1/1	49	splice_donor_variant&intron_variant	Fmo2
chr13_75683654_A_G	1/1	49	splice_acceptor_variant&intron_variant	LOC120096300
chr13_79509734_TC_T	1/1	43	frameshift_variant&stop_lost	LOC108352568
chr13_83283478_T_G;chr13_83283477_AT_A	1/1	49	frameshift_variant	Fcgr2a
chr13_83283481_A_G;chr13_83283481_A_AG	1/1	35	frameshift_variant	Fcgr2a
chr13_84029198_T_A	1/1	38	splice_donor_variant&intron_variant	Cd244
chr13_84359483_G_A	1/1	54	splice_acceptor_variant&intron_variant	Cd84
chr13_86116332_GGCTGTGCA_G	1/1	37	frameshift_variant	Olr1590
chr13_86164451_G_T	1/1	65	stop_gained	Olr1593
chr13_86394356_T_TA	1/1	38	frameshift_variant	Olr1602
chr13_90805895_TGTAA_T	1/1	39	splice_donor_variant&splice_region_variant&intron_variant	LOC108352580
chr13_92455965_GC_G	1/1	47	splice_acceptor_variant&splice_donor_variant&intron_variant	Acbd3
chr13_93947225_TAGCCGGAAATTACGTCA	1/1	32	splice_acceptor_variant&splice_region_variant&intron_variant&non_co	Degs1
chr13_95029289_A_G	1/1	64	start_lost	Taf1a
chr13_103187766_GT_G	1/1	55	frameshift_variant	Ints7
chr13_103187769_CT_C	1/1	38	splice_acceptor_variant&splice_donor_variant&intron_variant	Ints7
chr13_103189728_A_AT	1/1	41	frameshift_variant	Ints7
chr13_103189733_AT_A	1/1	58	frameshift_variant	Ints7

wkyn_high_impact_final

chr13_103200364_AG_A	1/1	65	frameshift_variant	Ints7
chr14_644257_CT_C	1/1	36	frameshift_variant	Vom2r70
chr14_646984_T_TGATA	1/1	41	frameshift_variant&stop_gained	Vom2r70
chr14_649496_C_CAG	1/1	47	frameshift_variant	Vom2r70
chr14_649498_CGG_C	1/1	43	frameshift_variant	Vom2r70
chr14_1877997_AC_A	1/1	55	frameshift_variant	LOC108352688
chr14_2561217_GTCC_G	1/1	54	splice_acceptor_variant&splice_region_variant&intron_variant&non_co	LOC120096551
chr14_2563967_C_T	1/1	36	splice_acceptor_variant&intron_variant	LOC120096551
chr14_2565114_T_C	1/1	66	splice_acceptor_variant&intron_variant	LOC120096551
chr14_2577692_G_C	1/1	57	splice_donor_variant&intron_variant	Tgfbr3
chr14_4705459_G_A	1/1	45	splice_donor_variant&intron_variant	LOC102554737
chr14_5107431_TG_T	1/1	35	frameshift_variant	LOC102555488
chr14_5107986_C_T	1/1	58	stop_gained	LOC102555488
chr14_5568146_T_C;chr14_5568146_T_TGTC	1/1	40	frameshift_variant	Dspp
chr14_5570247_A_T	1/1	54	stop_gained	Dspp
chr14_9063090_GT_G	1/1	46	frameshift_variant&splice_region_variant	Plac8
chr14_9063094_T_C;chr14_9063093_CT_C	1/1	48	splice_acceptor_variant&splice_donor_variant&intron_variant	Plac8
chr14_10219100_A_G	1/1	59	splice_donor_variant&intron_variant	LOC120096569
chr14_14015745_A_T	1/1	59	stop_gained	RGD1561226
chr14_14168500_T_A	1/1	55	stop_gained	RGD1561977
chr14_14459336_TC_T	1/1	56	frameshift_variant	RGD1562932
chr14_14461052_GA_G	0/1	54	frameshift_variant	RGD1562932
chr14_16514576_T_C	1/1	56	splice_donor_variant&intron_variant	LOC120096579
chr14_17442328_AT_A;chr14_17442328_A_AT	1/1	50	frameshift_variant	Rassf6
chr14_20955365_TA_T	1/1	53	frameshift_variant	LOC689798
chr14_21492968_T_C	1/1	34	splice_donor_variant&intron_variant	Tmprss11f
chr14_35802189_G_GTCCAC	1/1	55	frameshift_variant	LOC102550273
chr14_38550555_A_T	1/1	64	stop_gained	Guf1
chr14_44791674_TAC_T	1/1	54	frameshift_variant	LOC498369
chr14_45397279_G_A	1/1	39	stop_gained	Slc25a52
chr14_50877399_T_C	1/1	56	stop_lost	LOC120096514
chr14_69146094_G_T	1/1	50	stop_gained	Bod1l1
chr14_69457499_G_A	1/1	45	stop_gained	LOC120096627
chr14_76548194_A_G	1/1	31	splice_acceptor_variant&intron_variant	Zfyve28
chr14_76633208_C_CTTTTAAAA	1/1	48	frameshift_variant&stop_gained	Poln
chr14_80019968_C_G	1/1	56	splice_acceptor_variant&intron_variant	Emid1
chr14_80691161_A_T	1/1	61	splice_donor_variant&intron_variant	LOC120096651
chr14_83407278_T_C	1/1	57	splice_donor_variant&intron_variant	LOC102557304
chr14_95032602_AGGTGGCTAAAACTCTGT_	1/1	44	splice_donor_variant&splice_region_variant&intron_variant&non_codin	LOC102548772
chr14_95033571_A_C	1/1	57	stop_lost	LOC102548772

wkyn_high_impact_final

chr14_95254582_GGCAGCAGCGGCGGCAG(	1/1	32	frameshift_variant&splice_acceptor_variant&splice_region_variant&intr	Peli1
chr14_96089663_T_TG	1/1	50	frameshift_variant	LOC120096523
chr14_102558392_T_C	1/1	58	splice_donor_variant&intron_variant	LOC102550342
chr14_104565085_A_G	1/1	62	start_lost	Psme4
chr15_10401394_ACT_A	1/1	48	frameshift_variant	LOC498453
chr15_13885442_G_T	1/1	62	splice_donor_variant&intron_variant	LOC120096925
chr15_15164495_A_C	1/1	57	splice_donor_variant&intron_variant	LOC120097098
chr15_16398931_C_CA	1/1	31	frameshift_variant	Cfap20dc
chr15_17764206_GA_G	0/1	40	frameshift_variant	LOC120093102
chr15_19559319_A_G	1/1	55	splice_donor_variant&intron_variant	LOC108352896
chr15_20372973_T_G	1/1	62	splice_acceptor_variant&intron_variant	LOC120096953
chr15_27921767_TCTC_T	1/1	41	splice_donor_variant&splice_region_variant&intron_variant	Lrp10
chr15_28086287_CTTTTTTTTTTTTT_C;chr15_	1/1	44	splice_donor_variant&splice_region_variant&intron_variant	Psmb11
chr15_28155856_A_G	1/1	60	start_lost	Lmln2
chr15_28753608_C_T	1/1	62	splice_donor_variant&intron_variant	LOC108352925
chr15_29836934_T_A	0/1	44	stop_gained	Mcpt1l3
chr15_29859434_A_G;chr15_29859434_A_T	0/1	31	stop_gained	Mcpt9
chr15_37450774_GAAAA_G;chr15_37450774_	1/1	40	frameshift_variant	Neil2
chr15_38625050_A_G	1/1	63	splice_donor_variant&intron_variant	Msra
chr15_39004159_AC_A	1/1	50	frameshift_variant	Kif13b
chr15_39421848_CT_C	1/1	57	splice_acceptor_variant&splice_donor_variant&intron_variant	Fzd3
chr15_48329897_T_C	1/1	58	splice_acceptor_variant&intron_variant	LOC120097036
chr15_51177414_A_G	1/1	63	splice_donor_variant&intron_variant	LOC102549099
chr15_51361743_A_G	1/1	59	splice_donor_variant&intron_variant	LOC103690313
chr15_52407161_A_C	1/1	52	splice_donor_variant&intron_variant	Lacc1
chr15_53960157_A_G	1/1	58	splice_acceptor_variant&intron_variant	LOC120097049
chr15_54944648_T_C	1/1	57	splice_donor_variant&intron_variant	Elf1
chr15_64832295_T_G	1/1	52	splice_donor_variant&intron_variant	LOC103693748
chr15_78475395_G_A	1/1	67	splice_donor_variant&intron_variant	LOC120097061
chr15_90824732_G_A	1/1	61	splice_donor_variant&intron_variant	LOC102550532
chr15_98753670_C_CT	1/1	39	splice_acceptor_variant&intron_variant	Dock9
chr15_100055573_A_G	1/1	46	splice_donor_variant&intron_variant	Tmtc4
chr16_5587655_CT_C	1/1	42	frameshift_variant	LOC108348453
chr16_6367792_GGAGCCTGAGCCCCTGCCA	1/1	35	frameshift_variant&splice_acceptor_variant&splice_region_variant&intr	Nisch
chr16_7943455_T_A	1/1	51	splice_donor_variant&intron_variant	LOC102547142
chr16_9246082_AG_A	1/1	40	frameshift_variant	Gdf10
chr16_9246084_TGC_T	1/1	35	frameshift_variant	Gdf10
chr16_11551808_A_AC	1/1	58	splice_donor_variant&intron_variant	LOC100362043
chr16_14076193_T_TA;chr16_14076193_T_A	1/1	36	frameshift_variant	LOC683469
chr16_14076392_G_T	1/1	33	stop_gained	LOC683469

wkyn_high_impact_final

chr16_15841687_A_G	1/1	55	splice_acceptor_variant&intron_variant	LOC120097391
chr16_17707262_G_T	1/1	63	splice_acceptor_variant&intron_variant	LOC108348385
chr16_17717345_A_G	1/1	53	splice_acceptor_variant&intron_variant	LOC108348385
chr16_18423572_G_A	1/1	62	splice_donor_variant&intron_variant	LOC108348388
chr16_23046785_T_C	1/1	63	splice_acceptor_variant&intron_variant	Npy1r
chr16_25434019_A_G	1/1	55	splice_donor_variant&intron_variant	LOC103693926
chr16_28110048_A_T	0/1	34	stop_lost	Ddx60
chr16_38406160_G_GA	1/1	63	frameshift_variant&splice_region_variant	Neil3
chr16_46831898_G_T	1/1	70	stop_gained	Tlr3
chr16_48838681_G_C	1/1	51	splice_acceptor_variant&intron_variant	Zfp42
chr16_51246845_C_T	1/1	58	splice_acceptor_variant&intron_variant	LOC103693968
chr16_56463340_C_A	1/1	56	stop_gained	LOC100910088
chr16_61124455_G_T;chr16_61124452_ACA	1/1	63	splice_donor_variant&splice_region_variant&intron_variant&non_codin	LOC120097498
chr16_66470235_CA_C	1/1	36	frameshift_variant&start_lost	Letm2
chr16_69917639_G_A	1/1	54	splice_acceptor_variant&intron_variant	Nek5
chr16_69938390_T_G	1/1	60	stop_lost	Nek5
chr16_70613202_AT_A	1/1	33	frameshift_variant	Defb5
chr16_70994240_A_AC	1/1	48	frameshift_variant	Map1lc3b2
chr16_74637485_A_C	1/1	55	splice_donor_variant&intron_variant	Kbtbd11
chr17_5101091_AC_A	1/1	55	frameshift_variant	RGD1559908
chr17_10121570_A_G	1/1	61	stop_lost&splice_region_variant	RGD1566033
chr17_12335630_G_A	1/1	68	splice_donor_variant&intron_variant	Auh
chr17_13787615_C_A	1/1	55	splice_donor_variant&intron_variant	S1pr3
chr17_14662430_TCAGG_T	1/1	37	frameshift_variant	LOC100910585
chr17_14762809_G_A	1/1	64	stop_gained	LOC120097802
chr17_14847850_A_T	1/1	55	stop_gained	LOC102549716
chr17_15242997_A_C	1/1	46	stop_lost	LOC100361933
chr17_16062924_TG_T;chr17_16062924_T_T	1/1	57	frameshift_variant	Ptpdc1
chr17_17956463_T_C	1/1	60	splice_acceptor_variant&intron_variant	LOC120097853
chr17_18059988_C_CTAAGA	1/1	63	splice_acceptor_variant&intron_variant	Cap2
chr17_23771438_GAAGT_G	1/1	44	frameshift_variant	C17h6orf52
chr17_27350135_A_G	1/1	58	splice_donor_variant&intron_variant	LOC100910628
chr17_30935194_G_A	1/1	58	splice_donor_variant&intron_variant	Serpinb6a
chr17_31180743_T_TCTAC	1/1	33	frameshift_variant	Serpinb6e
chr17_31180744_AG_A	1/1	31	frameshift_variant	Serpinb6e
chr17_31378812_T_C	1/1	70	stop_lost	RGD1562844
chr17_31443112_A_AT	1/1	61	frameshift_variant	Serpinb9
chr17_33624454_C_CATGCAGGTAAGTGCA	1/1	54	splice_donor_variant&intron_variant	Exoc2
chr17_36176341_T_G	1/1	59	splice_donor_variant&intron_variant	LOC120097904
chr17_37205830_A_G	1/1	55	splice_acceptor_variant&intron_variant	Prl7b1

wkyn_high_impact_final

chr17_37708511_C_T	1/1	58	splice_donor_variant&intron_variant	Prl3a1
chr17_40072969_AAGACTCGAGTATGAAG	1/1	33	splice_acceptor_variant&splice_donor_variant&splice_region_variant&i	Mrs2
chr17_42798765_C_T	1/1	65	splice_donor_variant&intron_variant	LOC100360950
chr17_45199218_TA_T	1/1	54	frameshift_variant	Gpr141
chr17_53711790_A_G	1/1	41	splice_donor_variant&intron_variant	LOC120097865
chr17_57718122_C_A	1/1	59	splice_acceptor_variant&intron_variant	LOC103690071
chr17_57987118_G_GA	1/1	42	frameshift_variant	Anlnl1
chr17_61411208_T_G	1/1	49	splice_donor_variant&intron_variant	LOC103690178
chr17_71295911_T_G	1/1	58	splice_acceptor_variant&intron_variant	LOC102547255
chr17_71833354_C_T;chr17_71833354_C_CG	1/1	56	frameshift_variant	Usp6nl
chr17_72996564_T_C	1/1	58	splice_acceptor_variant&intron_variant	LOC108348589
chr17_72997189_CT_C	1/1	43	splice_acceptor_variant&intron_variant	LOC108348589
chr17_74505598_G_A	1/1	56	stop_gained	Fam107b
chr17_76002639_C_T	1/1	62	splice_donor_variant&intron_variant	LOC102555562
chr17_85355162_T_TA	1/1	59	frameshift_variant	Potec
chr18_405692_C_CT	1/1	48	frameshift_variant	LOC120098303
chr18_1699584_G_GTGAGAAGAC	1/1	44	splice_donor_variant&intron_variant	Snrpd1
chr18_3628677_G_T	1/1	62	splice_donor_variant&intron_variant	Lama3
chr18_5428625_A_G	1/1	61	splice_donor_variant&intron_variant	LOC108348847
chr18_12015362_TC_T	1/1	39	frameshift_variant	LOC102551486
chr18_18586629_T_A	1/1	56	splice_acceptor_variant&intron_variant	LOC102547510
chr18_18608420_T_C	1/1	43	splice_acceptor_variant&intron_variant	LOC102547510
chr18_23466245_AG_A	1/1	50	splice_acceptor_variant&splice_donor_variant&intron_variant	Wdr33
chr18_23510970_C_CG	1/1	31	frameshift_variant&splice_region_variant	Wdr33
chr18_23510987_TG_T	1/1	33	frameshift_variant	Wdr33
chr18_23518097_AC_A	1/1	37	frameshift_variant	Wdr33
chr18_23518104_AG_A	1/1	48	splice_acceptor_variant&splice_donor_variant&intron_variant	Wdr33
chr18_23526231_GC_G	1/1	48	frameshift_variant	Wdr33
chr18_50441999_G_T	1/1	56	stop_gained	LOC100174910
chr18_56111544_A_T	1/1	60	stop_lost	Spink10
chr18_58821330_TG_T	1/1	47	frameshift_variant&splice_region_variant	Alpk2
chr18_58821333_TC_T	1/1	49	splice_acceptor_variant&splice_donor_variant&intron_variant	Alpk2
chr18_59589991_C_T	1/1	52	stop_gained	Ccbe1
chr18_64132883_T_C	1/1	58	splice_acceptor_variant&intron_variant	Poli
chr18_67962739_ATCTT_A	1/1	51	frameshift_variant	LOC120098236
chr18_67962754_G_A	1/1	61	stop_gained	LOC120098236
chr18_72542909_A_AAC	1/1	34	frameshift_variant	LOC120098177
chr18_73412441_T_A	1/1	63	stop_lost	Mrps21l
chr18_80610351_C_CGT;chr18_80610351_C	1/1	43	frameshift_variant	RGD1560813
chr18_80610352_C_CCATG;chr18_80610352_	1/1	55	frameshift_variant	RGD1560813

wkyn_high_impact_final

chr18_83789757_A_C	1/1	55	stop_lost	LOC102549410
chr19_169497_T_G	1/1	67	stop_gained	Ces2e
chr19_169528_G_GA	1/1	58	frameshift_variant	Ces2e
chr19_232362_A_G	1/1	55	splice_acceptor_variant&intron_variant	LOC120098497
chr19_10658213_A_G	1/1	55	splice_acceptor_variant&intron_variant	LOC120098571
chr19_14886730_A_G	1/1	55	splice_acceptor_variant&intron_variant	LOC120098593
chr19_19799311_C_CT	1/1	38	splice_acceptor_variant&intron_variant	LOC102551515
chr19_20600525_A_C	1/1	49	stop_lost	Abcc12
chr19_22818504_C_T	0/1	43	splice_acceptor_variant&intron_variant	LOC120098581
chr19_24754950_AC_A	1/1	43	splice_acceptor_variant&splice_donor_variant&intron_variant	Mgat4d
chr19_29149555_C_A	1/1	42	stop_gained	Reeld1
chr19_32910880_ATGCGGCT_A	1/1	46	frameshift_variant	LOC108348882
chr19_32967515_G_GTGAGTACACA	1/1	55	splice_donor_variant&intron_variant	LOC102548129
chr19_32993816_T_C	1/1	60	splice_acceptor_variant&splice_donor_variant&intron_variant	Ces3a
chr19_32993911_A_AC	1/1	48	frameshift_variant&splice_region_variant	Ces3a
chr19_32996164_C_CAATCCTCACAGAGA	1/1	45	frameshift_variant	Ces3a
chr19_32998854_T_TCAGATGGCTG	1/1	41	frameshift_variant	Ces3a
chr19_33000099_G_A	1/1	64	splice_donor_variant&intron_variant	Ces3a
chr19_33000522_G_A	1/1	57	stop_gained	Ces3a
chr19_33582413_A_AC	1/1	55	frameshift_variant	Carmil2
chr19_33737484_C_CTT	1/1	31	frameshift_variant	Cenpt
chr19_34947610_G_GA	1/1	54	frameshift_variant	Vps4a
chr19_36880765_AACAG_A;chr19_36880765	1/1	31	frameshift_variant	Zfhx3
chr19_38754591_AAGCTACACGGCCAAGGG	1/1	44	frameshift_variant	LOC108348937
chr19_39656067_G_GA	1/1	65	splice_donor_variant&intron_variant	Ctrb1
chr19_40281089_A_G	1/1	64	splice_acceptor_variant&intron_variant	LOC120098565
chr19_45316276_C_T	1/1	62	stop_gained	LOC120098619
chr19_45316388_TCCCTAAC_T	1/1	31	frameshift_variant	LOC120098619
chr19_49989121_TCTTTGTAATGATAATACA	1/1	30	frameshift_variant&stop_lost	Ca5a
chr19_53972554_G_GC	1/1	56	frameshift_variant	Kcnk1
chr19_54439156_CATGTA_C	1/1	55	frameshift_variant	Tarbp1
chr19_54440528_AG_A	1/1	51	frameshift_variant	Tarbp1
chr20_412608_TA_T;chr20_412608_T_TA	1/1	51	frameshift_variant	Olr1683
chr20_1132768_A_C	1/1	56	stop_gained	Olr1876
chr20_1133260_G_GT	1/1	46	frameshift_variant	Olr1876
chr20_1133262_GA_G	1/1	47	frameshift_variant	Olr1876
chr20_1173314_TAGAAATAGGAG_T	1/1	38	frameshift_variant	Olr1731
chr20_1173787_G_GA	1/1	49	frameshift_variant	Olr1731
chr20_2605541_GC_G	1/1	48	splice_acceptor_variant&splice_donor_variant&intron_variant	RT1-T18
chr20_2670748_A_G	1/1	60	start_lost	RT1-S3

wkyn_high_impact_final

chr20_2774461_T_TG	1/1	54	frameshift_variant	RT1-T24-1
chr20_3074969_GA_G	1/1	45	frameshift_variant	Gtf2h4
chr20_3083973_AC_A	1/1	33	frameshift_variant	Vars2
chr20_3084008_CG_C	1/1	38	splice_acceptor_variant&splice_donor_variant&intron_variant	Vars2
chr20_3084035_CT_C	1/1	31	splice_acceptor_variant&splice_donor_variant&intron_variant	Vars2
chr20_3085782_CCT_C	1/1	42	frameshift_variant&splice_acceptor_variant&splice_donor_variant&intro	Vars2
chr20_3317239_A_T	1/1	45	stop_gained	RT1-CE13
chr20_3519950_GT_G	1/1	32	frameshift_variant	RT1-CE3
chr20_3539421_ACATCAATTCCCAT_A;chr20_	1/1	34	frameshift_variant	RT1-CE2
chr20_3651432_C_CG	1/1	31	frameshift_variant	Aif1
chr20_3679891_G_T;chr20_3679891_G_GCC	1/1	61	frameshift_variant	Bag6
chr20_3745797_CA_C	1/1	51	frameshift_variant	Ly6g6e
chr20_4161324_G_A;chr20_4161324_G_GC	1/1	54	frameshift_variant	Notch4
chr20_4236230_A_G	1/1	55	start_lost	RGD1624210
chr20_4312528_C_T	1/1	40	stop_gained	Btnl7
chr20_4405501_T_TAGGAAGGGG;chr20_44	1/1	46	frameshift_variant	Btn3a2
chr20_4478777_C_T	1/1	49	stop_gained	Btnl3
chr20_4554105_C_A;chr20_4554105_C_T	1/1	37	stop_gained	RT1-Db1
chr20_4554286_C_T;chr20_4554286_C_CCT;c	1/1	34	frameshift_variant	RT1-Db1
chr20_4823141_AG_A	1/1	59	frameshift_variant	Rxrb
chr20_4950973_GACACACACACACACACACA	1/1	31	splice_acceptor_variant&conservative_inframe_deletion&splice_region_	Rgl2
chr20_5018976_A_G	1/1	63	splice_acceptor_variant&intron_variant	Phf1
chr20_5618970_T_C	1/1	63	stop_lost	Smim29
chr20_5619120_C_T	1/1	62	stop_gained&splice_region_variant	Smim29
chr20_7221527_TCAGGGACCCATGACTA_T	1/1	46	splice_donor_variant&splice_region_variant&intron_variant	Cpne5
chr20_7750251_AAGGTGCTTTATGGGTGTT	1/1	40	frameshift_variant&splice_acceptor_variant&splice_region_variant&intr	Cmtr1
chr20_9783770_GT_G	1/1	36	splice_acceptor_variant&splice_donor_variant&intron_variant	Cryaa
chr20_9785233_GT_G	1/1	54	frameshift_variant	Cryaa
chr20_10006542_A_G	1/1	65	splice_acceptor_variant&intron_variant	LOC120098852
chr20_10841581_A_G	1/1	56	splice_donor_variant&intron_variant	LOC120098855
chr20_10888819_G_GCCCCCAGCTGCTGTG	1/1	38	frameshift_variant	LOC690460
chr20_12602345_C_A	1/1	54	stop_gained	Slc5a4b
chr20_27962859_A_ACAGCAGCCCGTGG	1/1	49	frameshift_variant	LOC103694426
chr20_30891168_A_ACCATTCAACACC	1/1	53	splice_donor_variant&intron_variant	LOC103694421
chr20_31178460_TGATACA_T	1/1	40	splice_acceptor_variant&splice_region_variant&intron_variant	LOC102555143
chr20_32044821_T_C	1/1	60	splice_acceptor_variant&intron_variant	LOC120098877
chr20_37957456_C_A	1/1	48	splice_donor_variant&intron_variant	LOC120099008
chr20_41932518_TC_T	1/1	49	frameshift_variant	LOC120098890
chr20_46916016_G_A	1/1	55	splice_donor_variant&intron_variant	Pdss2
chr20_48355045_A_T	1/1	57	splice_donor_variant&intron_variant	LOC102555236

wkyn_high_impact_final

chr20_48355046_C_G	1/1	58	splice_donor_variant&intron_variant	LOC102555236
chr20_54415346_C_CCT	1/1	55	frameshift_variant	LOC103694460
chrX_13059480_A_T	1/1	34	splice_donor_variant&intron_variant	LOC102551428
chrX_15097391_A_AC	1/1	41	frameshift_variant	RGD1566093
chrX_20994756_A_G	1/1	39	splice_acceptor_variant&intron_variant	Huwe1
chrX_23906102_T_TA;chrX_23906102_T_TAA	1/1	48	frameshift_variant	LOC120099331
chrX_42216202_AT_A	1/1	34	frameshift_variant	Ptges3l1
chrX_56862093_T_C	1/1	30	stop_lost	RGD1559970
chrX_59012229_GT_G	1/1	30	frameshift_variant	Klhl15
chrX_81793967_TG_T	1/1	31	splice_acceptor_variant&splice_donor_variant&intron_variant	Cpxcr1
chrX_81793969_G_A	1/1	30	splice_acceptor_variant&splice_donor_variant&intron_variant	Cpxcr1
chrX_105960903_TA_T	1/1	36	frameshift_variant	Acsl4
chrX_105960932_AG_A	1/1	36	frameshift_variant	Acsl4
chrX_107573686_T_C	0/1	32	splice_donor_variant&intron_variant	RGD1566355
chrX_130032962_C_T;chrX_130032962_CT_C	1/1	51	splice_donor_variant&intron_variant	LOC103690083
chrX_139593081_G_A	1/1	32	stop_gained	Cdr1
chrY_14482687_A_G	0/1	51	splice_acceptor_variant&intron_variant	LOC103694782
